# Validation and Automation of a High-Throughput Multitargeted Method for Semiquantification of Endogenous Metabolites from Different Biological Matrices Using Tandem Mass Spectrometry

**DOI:** 10.3390/metabo8030044

**Published:** 2018-08-05

**Authors:** Jatin Nandania, Gopal Peddinti, Alberto Pessia, Meri Kokkonen, Vidya Velagapudi

**Affiliations:** 1 Metabolomics Unit, Institute for Molecular Medicine Finland (FIMM), University of Helsinki, HiLIFE, Tukholmankatu 8, Biomedicum 2U, 00290 Helsinki, Finland; jatinnandania@gmail.com (J.N.); alberto.pessia@helsinki.fi (A.P.); meri.kokkonen@gmail.com (M.K.); 2 Roche Diagnostics GmbH, Nonnenwald 2, 82377 Penzberg, Germany; 3 Computational Systems Medicine group, University of Helsinki, 00290 Helsinki, Finland; cugopal@gmail.com; 4 Technical Research Center of Finland, P.O. Box 1000, 02044 Espoo, Finland; 5 Network Pharmacology for Precision Medicine Group, University of Helsinki, 00290 Helsinki, Finland; 6 Finnish Customs Laboratory, Tekniikantie 13, 02150 Espoo, Finland

**Keywords:** high-throughput, targeted, semiquantitation, metabolomics, LC-MS, multianalyte method, validation, cross-platform comparability, automation, biomarkers

## Abstract

The use of metabolomics profiling to understand the metabolism under different physiological states has increased in recent years, which created the need for robust analytical platforms. Here, we present a validated method for targeted and semiquantitative analysis of 102 polar metabolites that cover major metabolic pathways from 24 classes in a single 17.5-min assay. The method has been optimized for a wide range of biological matrices from various organisms, and involves automated sample preparation and data processing using an inhouse developed R-package. To ensure reliability, the method was validated for accuracy, precision, selectivity, specificity, linearity, recovery, and stability according to European Medicines Agency guidelines. We demonstrated an excellent repeatability of retention times (CV < 4%), calibration curves (R^2^ ≥ 0.980) in their respective wide dynamic concentration ranges (CV < 3%), and concentrations (CV < 25%) of quality control samples interspersed within 25 batches analyzed over a period of one year. The robustness was demonstrated through a high correlation between metabolite concentrations measured using our method and the NIST reference values (R^2^ = 0.967), including cross-platform comparability against the BIOCRATES AbsoluteIDQp180 kit (R^2^ = 0.975) and NMR analyses (R^2^ = 0.884). We have shown that our method can be successfully applied in many biomedical research fields and clinical trials, including epidemiological studies for biomarker discovery. In summary, a thorough validation demonstrated that our method is reproducible, robust, reliable, and suitable for metabolomics studies.

## 1. Introduction

Metabolomics has a great influence on many disciplines, as metabolites are intermediates or end products of cellular functions. Hence, metabolomics can be used as a powerful tool in understanding, diagnosing, and managing different pathophysiological conditions. It is therefore essential to be able to identify and measure metabolites from different biological matrices [1]. Although global metabolomics has been widely used in discovery studies for understanding cellular responses to normal and abnormal biological conditions, targeted metabolomics has more advantages for addressing biological questions in a more hypothesis-driven manner than global untargeted metabolomics [2]. Furthermore, targeted metabolomics can quantify metabolites that are low in abundance, which are difficult to assess using an untargeted approach.

An appropriate sample pretreatment is required to obtain reproducible and high-quality quantitative data in targeted metabolomics. However, metabolites are present in a wide dynamic range with great diversity in physicochemical properties in the biological matrices [3]. Recent advancements in extraction techniques and automated approaches for sample preparation have partially satisfied the demands of targeted metabolomics. However, there are still many outstanding challenges, such as the matrix effect and laboratory-to-laboratory variations associated with sample preparation. Hence, standardization of sample preparation is a fundamental requirement in metabolomics studies [4].

Secondly, a robust analytical methodology is required for an accurate quantification of metabolites with good reproducibility over an extended period of time [5]. Molecular diversity is a major problem that hinders the separation of all preselected metabolites in a single chromatographic run and the detection of all separated metabolites with a minimum technical variation [6]. Tandem mass spectrometry (MS) is a technique used predominantly due to its high sensitivity and high throughput for the detection of metabolites. A combination of MS and separation techniques is used to increase the sensitivity and reliability of analytical methods for the analysis of metabolites from complex biological matrices [7]. In addition, the latest developments in triple quadrupole instrumentation strengthened the possibilities to develop multianalyte methods in a single injection that yield reliable and quantitative data [8]. Several analytical methods have also been developed for semiquantitative measurement of large number of metabolites in a single run [9,10,11,12,13,14,15].

Even though liquid chromatography-MS (LC-MS) is a method of choice in targeted metabolomics, obtaining an accurate quantification and long-term data reproducibility remains an analytical challenge. This is due to limitations such as matrix effect, MS performance drift, and LC column contamination and aging [16]. Finally, although instrument vendor software for data processing provides some crucial functions, such as peak integration (rendering the data from high-throughput metabolomics experiments into numerical values that represent metabolite concentrations), the lack of automation in downstream data processing and quality control remains a major bottleneck in high-throughput analyses. Thus, an efficient pipeline is necessary to enable rapid, accurate, and standardized processing of these data. Such a pipeline should facilitate automated analyses, while at the same time allowing the user to fine-tune the parameters for accurate data processing. Taken together, there is a need for development and validation of standardized, robust, and quantitative methods for large-scale targeted metabolomics studies in a high-throughput manner to minimize the bias associated with sample preparation and the analytical technique used.

We have previously developed a robust, reproducible, and high-throughput targeted method for measuring 102 polar metabolites from various biological classes semiquantitatively in a single injection [17,18]. Furthermore, the method has been used in many biomedical and clinical studies for biomarker discovery [17,18,19,20,21,22,23,24,25,26,27,28,29,30,31,32,33,34,35,36,37,38,39]. The metabolites were selected according to the following criteria: have important roles in many biological processes, are known biomarkers in several diseases and technical feasibility for developing an analytical method that covers all the metabolites in a single assay. The selected metabolites come from 24 different classes (Table 1), covering a wide range of metabolic pathways. We created an inhouse metabolite database by manually curating all the available information (i.e., names, HMDB, PUBCHEM, KEGG Ids, chemical properties, reported normal and abnormal concentration ranges, links to their structures) from the Human Metabolome Database (HMDB). The selected metabolites were separated by Hydrophilic Interaction Liquid Chromatography (HILIC) and measured using triple quadrupole tandem MS. A detailed method description is provided in the supplementary text.

The primary objective of this work is to show the robustness of our previously developed analytical method through a thorough validation according to European Medicines Agency (EMA) guidelines. We also demonstrate the automation of tedious and manual data-processing tasks in high-throughput metabolomics analyses using an inhouse developed R-package. The R-package automates various corrections and normalization steps to convert the raw peak area data to molecular concentrations for each compound in each sample and also provides quality evaluation of the data and reduces the manual workload significantly.

## 2. Materials and Methods

### 2.1. Chemicals and Reagents

All metabolite standards were purchased from Sigma-Aldrich (St. Louis, MO, USA). Internal standards were ordered from Cambridge Isotope Laboratory. Inc. (Tewksbury, MA, USA). LC-MS-grade solvents, 2-proponol, acetonitrile, and methanol (HiPerSolv) were obtained from VWR International (Helsinki, Finland). Analytical-grade chemicals (formic acid, ammonium formate, and ammonium hydroxide) were obtained from Sigma-Aldrich. Deionized water (18 MΩ·cm at 25 °C) used for solution preparation was made using a Milli-Q water purification system (Bamstead EASYpure RoDi ultrapure water purification system, Thermo scientific, Waltham, OH, USA). Mouse tissues, including heart, liver, brain, spleen, and muscles were obtained from Innovative Research Laboratory (Novi, MI, USA). The whole blood, from which serum was prepared during method optimization and validation, was obtained from the Finnish Red Cross blood service (Helsinki, Finland). Cell samples were provided by our research collaborators. NIST Standard reference material (SRM) 1950 plasma was purchased from Sigma-Aldrich (Gillingham, UK).

### 2.2. Metabolite Extraction Protocol and Instrumentation

All metabolites were extracted, separated with HILIC (Acquity BEH amide, 2.1 × 100 mm, 1.7 µ), and analyzed with a Waters Xevo TQ-S triple quadrupole mass spectrometer using our previously published protocol [18]. The protocol for tissues and adherent cells was optimized for better recovery and chromatography and to cover a wide range of tissue and cell types with a single protocol. For tissue sample extractions, 90/10% ACN/H_2_O + 1% formic acid was used instead of 80/20% ACN/H_2_O + 1% formic acid during the second step of extraction. Additionally, during cell pellet sample extraction, 80/20% ACN/H_2_O + 1% formic acid was replaced with 90/10% ACN/H_2_O + 1% formic acid. After optimization, we used the tissue protocol for analysis of various biological matrices, such as heart, liver, placenta, brain, muscles, spleen tissues, dental carries, and *Drosophila* larvae (weight 10–20 ± 5 mg), *C. elegans* (2000–4000 worms/sample), dried blood spots (12–15 punched spots), and fecal samples (weight 20–50 ± 5 mg). The cell pellet protocol was used for all types of adherent cells (around 1 million cells/sample) and *E. coli* (3-day old bacterial lawns) and *S. cerevisiae* (OD_600nm_ ~ 2.2) samples. The biofluid protocol was used for all types of biofluids, such as blood, plasma, serum, cell culture supernatant, CSF, and urine (sample volume 50–100 µL).

### 2.3. Method Validation

Validation of the method was performed to verify various parameters and the reliability of the developed method for the analysis of a large number of samples. The method was validated according to EMA guidelines for bioanalytical method validation in terms of selectivity, specificity, linearity, accuracy, precision, extraction recovery, matrix effect, and stability [40]. In addition, we used pooled healthy human serum samples as internal quality control (QC) samples in all studies to correctly signal drift during sample runs and to improve confidence in the statistical data. QC samples at high, medium, and low (for serum) or high and low concentration levels (for tissues) were prepared by spiking a mixed standard solution in their respective homogenized biological matrices to perform all the method validation experiments. We performed the validation for commonly used biological samples in metabolomics analyses, such as biofluid (serum), tissue (liver, brain and spleen), and cell samples. An aqueous calibration curve was used to calculate the concentration values during the method validation. The instrument performance for response reproducibility and sensitivity was always verified by 6 consecutive injections of a medium concentration solution at the start of any experiment.

#### 2.3.1. Selectivity and Specificity

The selectivity and specificity for each metabolite were investigated using serum-spiked samples (N = 6) with known amount of standard. Chromatographic interferences from other endogenous compounds of the biological matrix at the retention time of the target analyte for a particular metabolite were verified. The chromatographic peaks from spiked samples were compared with the standards by the retention times and if required from their respective MRM spectra.

#### 2.3.2. Linearity, Accuracy, and Precision

To assess the linearity, accuracy, and precision, six replicates of spiked QC samples at high, medium, and low concentrations along with calibration curve standards were injected on three separate days. Calibration curve standards of 11 points were prepared via serial dilution and each calibration curve has over 1000-fold dynamic concentration range. The curve was plotted by using the peak area response ratios (standard/labeled standard) versus the concentrations of the individual metabolites. Each calibration curve was statistically evaluated and constructed using appropriate regression models, weighing factors and transformations. The accuracy was calculated as the measured value divided by the nominal value at each concentration level of the calibration curve standards in all three batches. Inter- and intrabatch variability was calculated by measuring coefficient of variation (%CV) at each QC concentration level.

#### 2.3.3. Recovery and Matrix Effect

The recovery efficiencies for each analyzed metabolite were determined by comparing analytical results from QC samples spiked with a standard mixture before and after extraction using different concentrations. The spike concentrations covered the calibration range. The matrix effect (percentage of ion suppression or enhancement of the MS signal) was determined by comparing the analytical response of the QC samples that were spiked after extraction with the analytical response of aqueous spiked samples (diluent spiked with respective concentrations of QCs). Since there were endogenous metabolites, we subtracted the endogenous concentrations from the samples that were spiked. This experiment was performed using 6 QC replicates.

#### 2.3.4. Stability of the Metabolites

Wet extract, freeze-thaw, and stock solution stability for all metabolites were determined to check the integrity of the analytes in solvents and in QC samples at different conditions. To determine the wet extract stability, six replicates of extracted QC samples were kept in the autosampler at 5 °C. The same samples in the same sequence were reinjected with freshly extracted QC samples and the results were compared.

Freeze-thaw stability was evaluated up to three cycles by freezing and thawing the spiked QC samples stored at –80 °C (for 12–16 h) and comparing the concentrations against the freshly thawed and spiked QC samples.

Long-term stock solution stability for metabolite stock solutions and intermediate solutions were checked by comparing the mean peak area of freshly prepared solutions with stored solutions at 4 °C. All stability experiments were performed with 6 replicates of QCs.

#### 2.3.5. Carryover

The carryover was evaluated by injecting the highest standard concentration (ULOQ) of the metabolites in the calibration curve followed by a series of blank injections and lowest standard concentration (LLOQ). The blank samples were evaluated for any signal at the retention time of particular metabolites and signal intensities of the blank samples were compared with the LLOQ samples. The acceptance criteria for carryover was set at 20% of the peak area corresponding to the LLOQ level as per the EMA guidelines for bioanalysis.

#### 2.3.6. QC Samples

Internal QC samples were prepared after separating serum from pooled healthy human blood samples. A volume of 350 µL of serum was aliquoted and stored at –80 °C after providing a lot number and QC number. The concentration of QC samples that were incorporated in batches during the metabolomics studies was calculated for all the metabolites along with the experimental samples. Average concentrations (μmol/L) and %CV of the QC samples were calculated for each metabolite. The data were saved along with QC lot numbers, batch name, and run date. The QC data were collected from six different lots for a period of 5.5 years (N = 539 replicates). An internal QC database has been maintained and used for quality checks.

#### 2.3.7. Comparison with Reference Material

To evaluate the performance of our semiquantitative method, commercially available standard-reference plasma (NIST SRM 1950) [41] was analyzed using our method (N = 8 replicates). The concentration values from the matched 17 metabolites were compared with the given standard reference values.

#### 2.3.8. Cross-Platform Comparison

To further evaluate the robustness and performance of our method, we performed a cross-platform comparison using two completely different analytical platforms: (1) the commercially available AbsoluteIDQ p180 targeted metabolomics assay kit using LC-MS/MS and (2) a nuclear magnetic resonance (NMR) platform. We sent our internal QC samples to the BIOCRATES Life Sciences AG (Innsbruck, Austria) (N = 3 replicates) and to the NMR Metabolomics Laboratory, School of Pharmacy, University of Eastern Finland (Kuopio, Finland) (N = 3 replicates). Our QC samples were extracted and analyzed as described previously for the AbsoluteIDQ p180 kit [42] and for the NMR analysis of small molecules [43]. We compared these results with the results obtained from our method (N = 4–5 replicates).

### 2.4. Statistical Analyses

To estimate the median concentration values (μmol/L) of the metabolites from the QC samples (N = 539), we fitted a linear mixed model with the MCMCglmm R-package [44] using an expanded parameter formulation and default settings. Observed data were assumed to be log-normally distributed and corrected for the six different QC lots. Credibility intervals (95%) of the median concentration values were computed from 20,000 samples of the posterior distribution. Error bars shown in the scatter plots are 95% confidence intervals, while the coefficient of determination R^2^ was obtained from the linear regression between the variables. Coefficient of variation (CV) percentages was calculated as a measure of variability. To automate the downstream processing of data produced by the instrument vendor software (TargetLynx, v4.1), we built a data-processing package called “Unlynx” in R statistical programming language. The R-package is available upon request.

### 2.5. Automated Data Processing

The “Unlynx” package parses the output of TargetLynx software (i.e., raw data containing the concentration values in PPB units) and produces a processed dataset in an Excel spreadsheet after performing a series of preprocessing operations.

The preprocessing steps included the following:

(i) Molecular weight normalization, in which the ppb values are normalized by the molecular weight of each compound, thereby converting the data from ppb units to µmoles.
(ii) Process efficiency correction for the semiquantification of metabolites without internal standards.
(iii) Normalization using dilution factor for specific sample type if dilution was needed.
(iv) Cell number normalization (for cell samples) to convert the concentration values per million cells.
(v) Calculation of mean, standard deviation, and relative standard deviation (RSD) of molecular concentrations (resulting from the previous steps) for each phenotypic group.
(vi) Outlier detection in each phenotypic group; if the concentration value of a compound in a sample is more than one or two standard deviations (SD) away from the mean of the phenotypic group, then it is marked as an outlier in the Excel data set in two different colors.
(vii) QC check by comparing the RSD of QC samples in the current dataset against the internal database of QC sample RSDs (based on interday RSDs recorded over one year).

## 3. Results and Discussions

### 3.1. Extraction Method Optimization

The primary objective of this work was to optimize and validate our previously published protocol for different types of biological matrices. For tissue samples (placenta, liver, heart, brain, spleen, and muscles), the sample volumes of the tissues and extraction solvent volumes were optimized to fit the concentrations of most of the metabolites within the linearity of calibration curve for reliable results. We observed that most of the metabolites could be semiquantified within the calibration curve range with 20 ± 5 mg of sample weight.

Furthermore, we optimized the protocol with extraction solvent for tissues and adherent cells. Some of the metabolites (in particular inositol, GABA, asymmetric dimethylarginine, symmetric dimethylarginine, spermidine, ribose-5-phosphate, and orotic acid) had poor separation and irreproducible chromatography. Interference of isobaric compounds with other metabolites was also observed due to poor separation. Thus, different compositions of the extraction solvent were assessed to achieve the acceptable chromatography. We observed that modification of acetonitrile content from 80% to 90% and applying longer equilibration time for the HILIC column yielded acceptable chromatography and also good separation for most of the metabolites.

We also optimized the extraction protocol for different sample types from various organisms (human, mouse, rat, dog, drosophila, nematode, yeast, bacteria), such as tissue types (spleen, pancreas, muscles, adipose, endometrium, testicles, lung), biofluids (plasma, blood, urine, cyst fluid, bile, CSF, saliva), cell types (adherent cells, cell suspension, bone marrow cells, extracellular vesicles, mitochondrial isolates, *E. coli*, and *S. cerevesiae*) and other sample types such as fecal samples, dried blood samples, dental carries, biofilm, drosophila (larvae and whole flies), and *C. elegans*.

**Figure 1:**
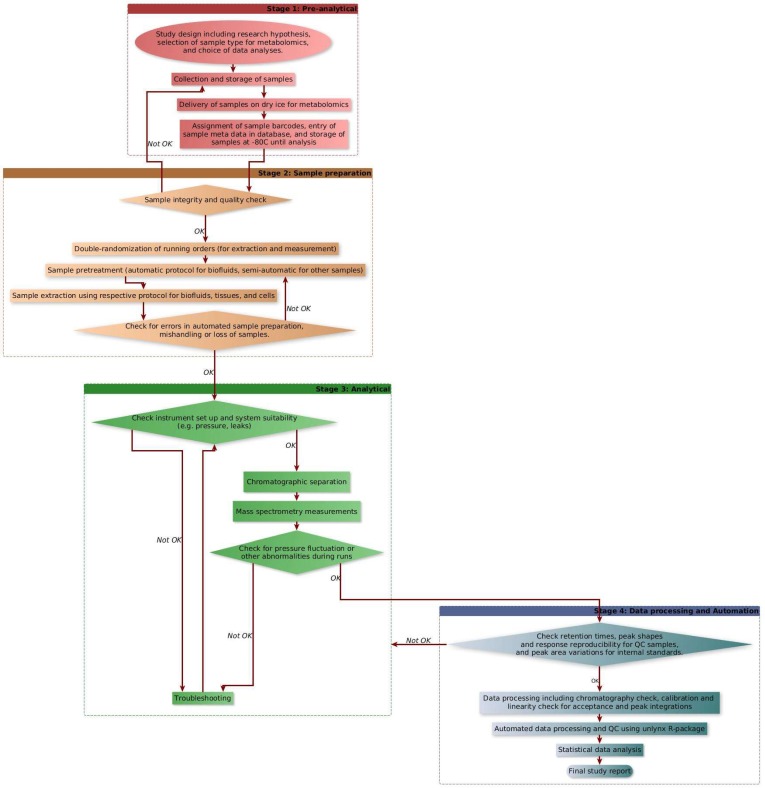
A flowchart describing preanalytical stage, sample treatment and analysis, analytical stage, automation, and quality checks.

A flowchart describing various stages of our workflow (preanalytical stage, sample analysis, analytical stage, data processing, and automation with quality checks) is shown in Figure 1.

### 3.2. Method Validation

#### 3.2.1. Selectivity and Specificity

There were no significant interference peaks from the matrix components in their respective retention-time windows, indicating the selectivity of the metabolites in our method. We repeated the injections for five times from all different serum samples and confirmed every peak to have eluted only from the target analyte, indicating that they are specific to their corresponding MRM transitions. The chromatograms for all the 102 metabolites of QC sample were given in Supplementary Figure S1.

#### 3.2.2. Linearity, Accuracy, and Precision

To cover a broad concentration range, when fitting the heteroscedastic calibration data, linear or quadratic regression models with appropriate weighing factors (1/x) and transformations (log-log) were used [45]. The coefficient of determination (R^2^) value for each metabolite was greater than 0.980 at their respective concentration range, except for some metabolites such as aspartate, uracil, 2-deoxyuridine sucrose, and chenodeoxycholic acid (likely due to their broad peak shapes and poor recovery at lower concentration, Table S1).

**Figure 2:**
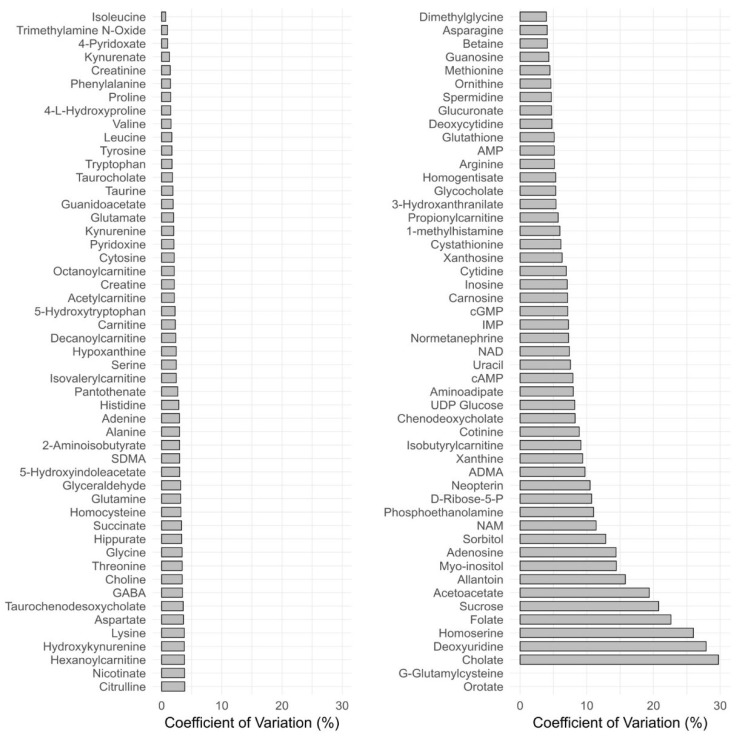
Interbatch variation of high concentrations of spiked quality control (QC) serum samples (N = 6) analyzed on 3 different days. Data not shown for g-glutamylcysteine and orotic acid (coefficient of variation (CV) > 30%).

The concentration precision for QC samples was calculated by measuring %CV at high, medium, and low concentration level of QCs (N = 6 replicates). In general, intra- and interday precision (CV) values were within 15% for 94 metabolites except acetoacetic acid, folic acid, sucrose, homoserine, 2-deoxyuridine, and cholic acid at high concentration (Figure 2). However, at low concentrations, more than 20% CV was observed for NAD and myo-inositol. This might be due to low recoveries of these compounds. Hence, these compounds cannot be measured reliably with this method even semiquantitatively.

#### 3.2.3. Recovery and Matrix Effect

**Figure 3:**
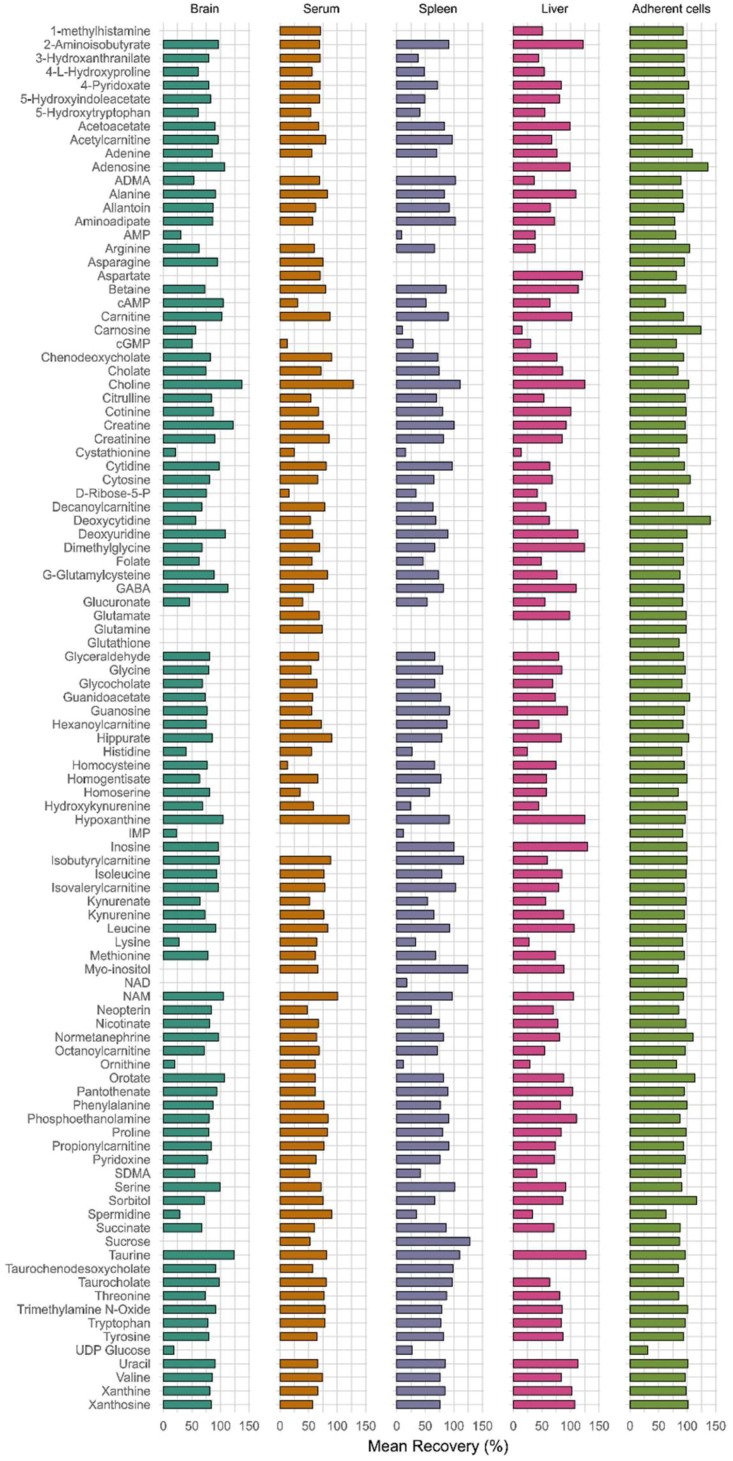
Percentage mean recoveries for all metabolites at low, medium, and high concentration levels of QCs (N = 6 at each level) spiked in serum, brain, spleen, liver, and adherent cells. Data not shown for metabolites with poor chromatography and irreproducible results at different concentration levels.

For 85–90 metabolites, recoveries were found to be between 50 to 120% with good repeatability at all three concentrations levels (low, medium, and high) in both biofluid (serum) and tissues (brain, liver, and spleen). However, compounds such as UDP-glucose, IMP, cGMP, d-ribose 5-phosphate, NAD, AMP, homocysteine, carnosine, and glutathione had recoveries less than 30% in serum. However, CV of recoveries at low, medium, and high concentration levels was within 25% except for cGMP in serum. Metabolites such as 1-methylhistamine, aspartate, glutamine, adenosine, and glutathione had recoveries over 120%. Histidine, ornithine, cystathionine, 3-OH-dl-kynurenine, carnosine, AMP, NAD, cGMP, IMP, and UDP-glucose had less than 30% recovery in some tissues types, indicating matrix effect or degradation (Figure 3). However, CV of repeatability at every concentration level was within 15% except for 1-methylhistamine, aspartate, glutamine, glutathione, NAD, and UDP-glucose in some tissues.

The matrix effect values were observed to be within the range of 0.6 to 1.8 (below 1 indicates ion suppression and above 1 indicates ion enhancement) for the metabolites in serum and tissues. The challenge of the matrix effect can be overcome by having individual isotope-labeled internal standards for each individual compound for true quantification. However, this is not practically possible for high-throughput metabolomics analyses. This is due to high costs and also because not all internal standards are commercially available. In our method, we selected 12 labeled internal standards (Table S1), which represent chemically similar classes for optimal correction. This is because the matrix effect was expected to be the same for an analyte and its labeled isotope analogue. The process efficiency percentages were calculated for the metabolites without internal standards. The analyte concentrations determined through the external calibration were divided with the total process efficiency values to correct the concentration values of the analyte in the given biological sample. The repeatability of the matrix effect in terms of CV was also less than 25% for most of the compounds. Reliable measurements are accordingly possible.

#### 3.2.4. Stability

**Figure 4:**
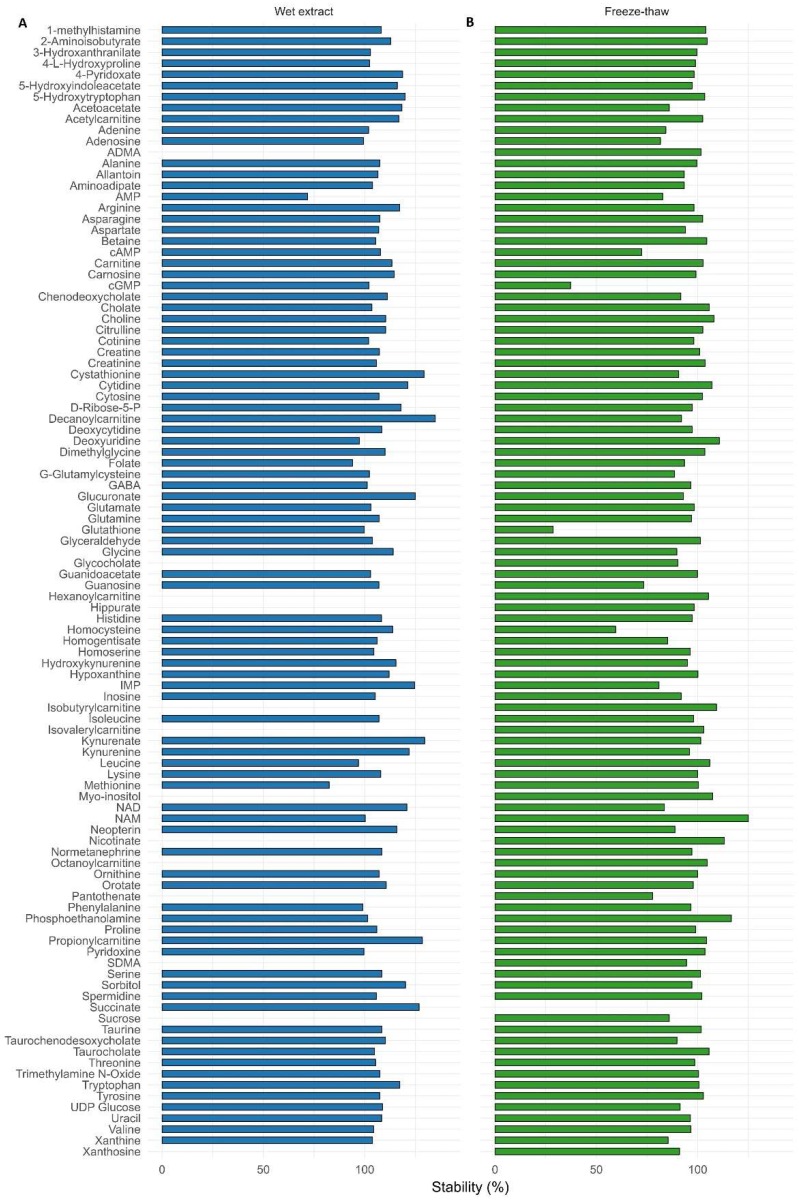
Percentage mean stability was calculated for three different concentrations (low, medium, and high) spiked in QC serum (N = 6 at each level) during (**A**) wet extract and (**B**) freeze-thaw stability for all the metabolites.

Some of the endogenous metabolites are not stable due to degradation or conversion reactions. Hence, the stabilities of all metabolites were assessed under different conditions. For wet extract stability, approximately 90% of the metabolites were stable (stabilities range between 85 and 115%) for 35 h at 5 °C in the autosampler (Figure 4A).

For freeze and thaw cycle stability, most of the metabolites were stable even after three freeze and thaw cycles, with the exception of cGMP, succinate, glutathione, and homocysteine (stability below 30%, Figure 4B). This information is particularly important for clinical studies, where samples are often thawed once or twice.

**Figure 5:**
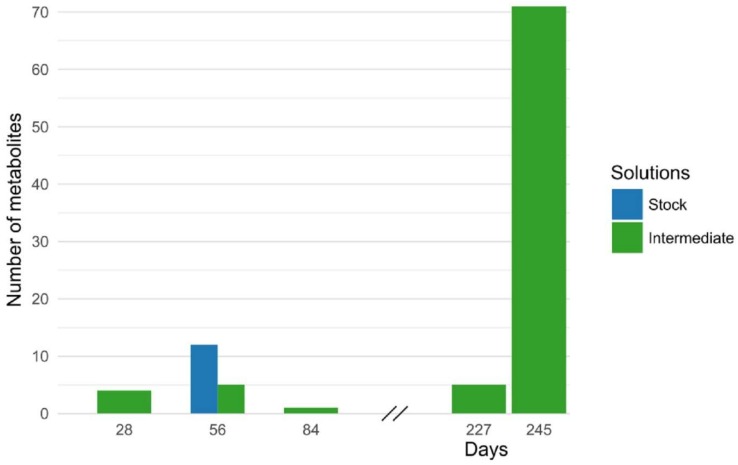
Bar graph representing the stability of stock solutions and intermediate solutions of metabolites that were used to prepare calibration curve standards during the analysis. First, the stability was determined at the intermediate concentration levels (green bar) and then at the stock level (blue bar) only for those metabolites, which were not stable at the intermediate concentration level.

To determine the stability of working solutions, we started evaluating the stability from intermediate solutions for all the metabolites. Most of the metabolites were stable for 245 days at intermediate concentration when stored at 4 °C. However, 16 metabolites had low stability at intermediate concentration; stabilities were thus determined at stock-level concentration for these compounds. We observed that the stock solutions were stable for 56 days except taurocholic acid, sucrose, UDP-glucose, and glutamine (Figure 5). Hence, these stock solutions were freshly prepared during the analysis. The stability of internal standards solutions was also assessed and they were stable for one year.

#### 3.2.5. Carryover

In general, for the majority of the analyte MRM channels neither a peak nor any interference in the blank samples was detected after injection of the metabolite standard with high concentration. For compounds such as spermidine, succinate, AMP, and IMP, carryover eluted constantly even after washing but was not significantly high. Other than these compounds, we can conclude that the column, needle, syringe, and seal washes were sufficient to avoid any intersample carryover.

#### 3.2.6. Reproducibility

**Figure 6:**
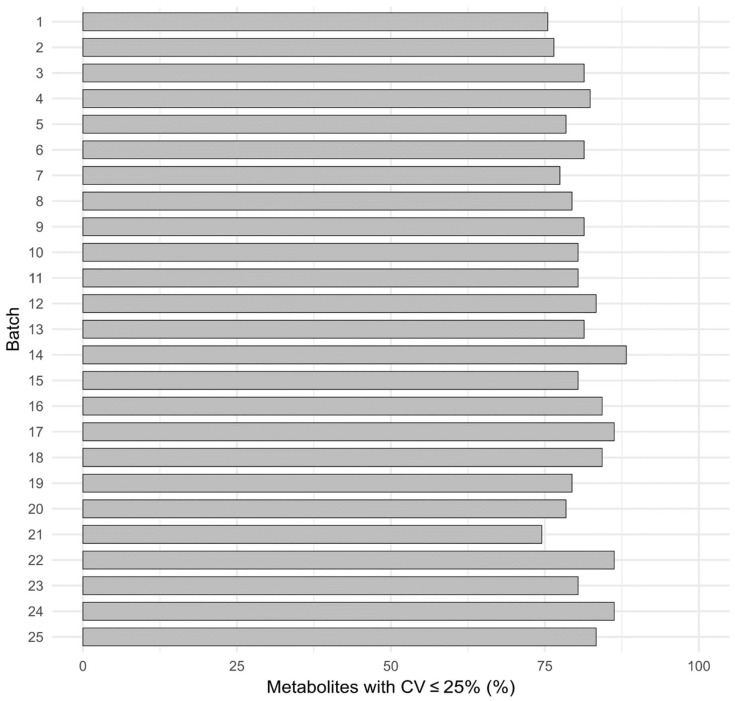
Percentage of metabolites with less than 25% CV values of QC concentrations in 25 different batches analyzed over a period of 1 year.

To ensure good quality of the data, internal QC samples were incorporated to a batch of samples and run after every tenth experimental sample. QC data were collected from 25 different batches that were performed during various metabolomics studies over a period of 1 year. Mean concentrations and %CV values of QC replicates within each batch were calculated for all the 25 batches. We observed that approximately 80 to 85% of the metabolites were always present within 25% of CV values (Figure 6). The higher %CV values for the remaining metabolites could be partially explained by low abundance in human serum, low recovery, or poor chromatography; these were consistently found to be below LLOQ within the 25 batches.

**Figure 7:**
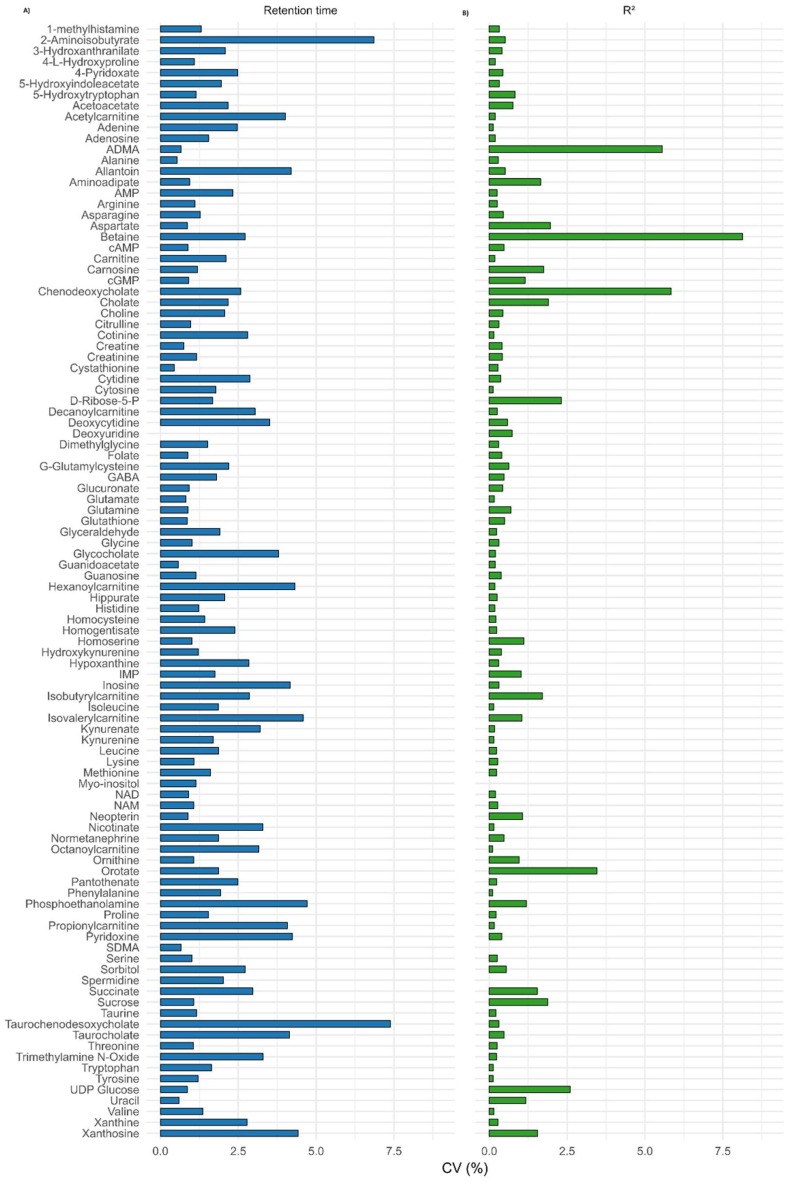
Reproducibility of (**A**) retention time of respective chromatographic peaks and (**B**) regression coefficient values (R^2^) from external calibration curve standards for each metabolite analyzed in 25 different batches over a period of 1 year.

In addition, %CV values for retention times and R^2^ values of calibration curves for each metabolite in all the 25 batches were calculated to verify the reproducibility. Based on these results, the repeatability was excellent except for a few compounds over a period of 1 year. No drifting effect for the retention times (CV < 4%) was observed, and excellent reproducibility was observed for R^2^ values of calibration curves (CV < 3%) (Figure 7). On the basis of these results, our method can be considered accurate, reliable, and reproducible.

#### 3.2.7. Quality Management

To obtain reproducible and accurate data, we set up a strict quality management and electronic lab notebook system. To reduce the bias from sample analysis, we always double-randomized the samples (i.e., one before the sample extraction step and one before injecting into the LCMS system across different phenotypes of the samples). For stabilization of response and retention time, we always verified a few runs of highest calibration level 11 before injecting the experimental samples. During the stabilization process, we also verified the chromatography, including peak shape, retention time, and response of all the metabolites. Any significant changes in the intensity, peak shape, retention time, and system pressure were thoroughly investigated and corrected by resolving the problems before injection of experimental samples.

**Figure 8:**
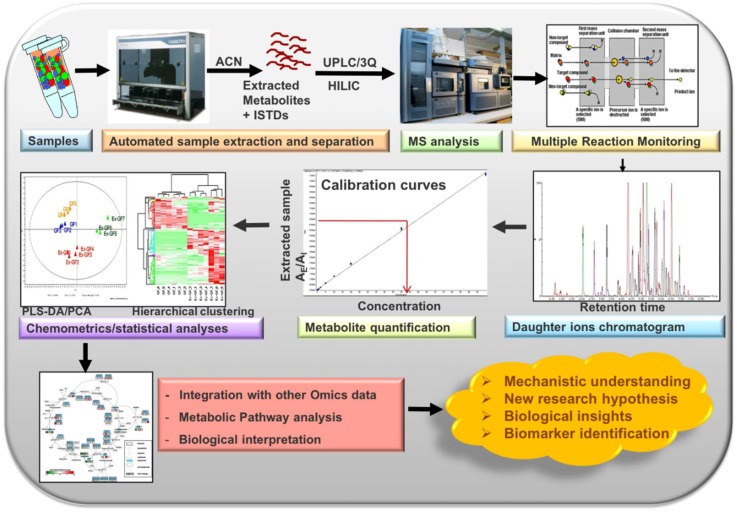
A typical workflow for high-throughput targeted metabolomics analysis.

To ensure the integrity of LCMS runs, QC samples were run at every tenth experimental sample and a blank sample at every fifth run during all the metabolomics studies within a batch. Furthermore, chromatography and response of QC samples (including chromatography of some metabolites and IS response variation) and blank runs were always verified after completion of the runs and before starting data processing. In case of any abnormality observed for particular samples, those samples were reinjected or reanalyzed. Only after passing these quality checks we proceeded further to process the data. This included verifying the accuracy of calibration curve standards, chromatography peak integrations, IS response variation, and verifying LLOQ and ULOQ for each sample for all metabolites within a batch. The high-throughput targeted metabolomics workflow is shown in Figure 8.

We collected concentration values (μmol/L) for our QC samples within metabolomics studies conducted over a period of 5.5 years from six different lots (N = 539 replicates). The median values of each metabolite together with a 95% credibility interval are presented in Table 1. These represent a reference level for a population of healthy adult individuals.

#### 3.2.8. Robustness and Cross-Platform Comparison

**Figure 9:**
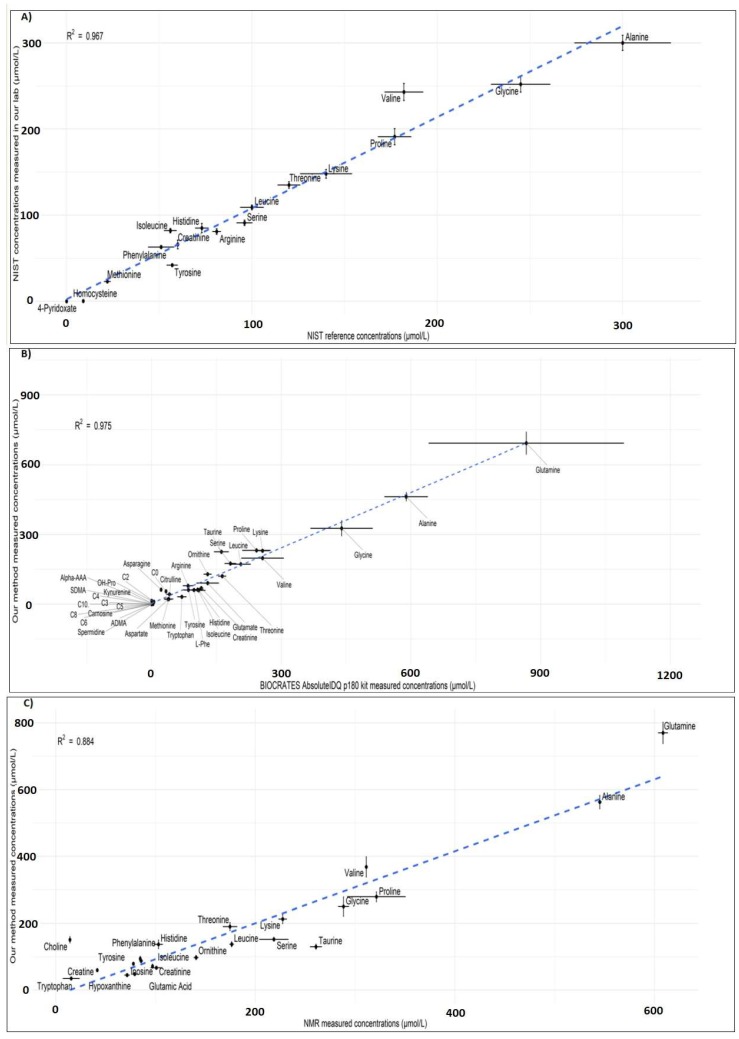
(**A**) Comparison of metabolite concentration reference values given in the NIST SRM 1950 plasma against our method. Comparison of metabolite concentrations in our QC samples measured between our method and (**B**) BIOCRATES AbsoluteIDQ p180 kit, and (**C**) nuclear magnetic resonance (NMR) analyses. Error bars represent 95% confidence intervals.

To verify the performance of our method, we analyzed the NIST standard reference material SRM 1950 plasma. The correlation coefficient for 17 matched metabolites between the given reference values and from our semiquantitative method was 0.967, indicating the high performance of our method (Figure 9A).

Furthermore, we verified cross-platform comparability. This was achieved by comparing metabolite concentrations analyzed using our method against two completely different analytical platforms (BIOCRATES AbsoluteIDQ p180 kit and NMR) in our QC samples. We obtained a high correlation coefficient for matched 38 metabolites measured using BIOCRATES AbsoluteIDQ p180 kit and our method (R^2^ = 0.975) (Figure 9B) and for matched 22 metabolites measured using NMR and our method (R^2^ = 0.884) (Figure 9C). These results demonstrate the robustness of our method.

### 3.3. Automated Data Processing

After the raw data processing using the instrument-coupled software (TargetLynx), an unstructured flat text file containing information such as sample ids, file name, and concentrations (PPB) for all metabolites is generated in a complex format. For example, in a single-batch run of 85 samples, after the data processing, the concentration values for all samples are separately obtained under each metabolite. This means that, if a data matrix of 85 samples × 100 metabolites is desired, each individual concentration value must be copied and pasted in another sheet in a tabular format. Then, the PPB values are to be converted into µm and corrected for process efficiencies and dilution factors (if any) and normalized with tissue weight or cell number depending on the sample type. Apart from these steps, as the samples are analyzed in a randomized manner, the next steps would include rearranging the experimental samples according to phenotypic group and separating the QC samples from the study samples. This manual data processing creates a ready-to-use data matrix for visualization and for downstream statistical analyses, but is tedious, time-consuming, and, more importantly, prone to errors.

To automate the manual data processing, we have implemented a software package “Unlynx” in R statistical language. This package takes the raw data produced by TargetLynx software as input and produces processed data into ready-to-use spreadsheets. We use Unlynx to covert the PPB values to µmol/L, µmol/g, or µmol/million cells by dividing with molecular weight of the respective metabolites and other correction factors (e.g., weight of tissues, number of cells, and dilution factor) appropriate for the sample type. In addition, mean concentrations and %CV of QC samples for all the metabolites are calculated and used to evaluate quality by comparing with the inhouse QC database constructed based on inter-day %CV. Furthermore, Unlynx retrieves LLOQ, ULOQ, and outlier values in each phenotypic group according to one and two SDs, retention time values, and R^2^ values for each metabolite. With the automated data processing, we reduced a two-day manual workload to a few minutes.

Thus, after processing the data using TargetLynx, we routinely exported all results for the automated data processing using Unlynx. Typically, the data resulting from such automated data processing undergoes more specialized data analyses (such as statistical hypotheses testing, classification, regression, and clustering) aimed at answering specific scientific questions related to the study design.

### 3.4. Applicability of the Method

We have applied our fully validated analytical methodology in various international and national biomedical research projects, epidemiological studies, clinical studies including dietary interventions, and clinical trials. We have successfully implemented our technology in the following research fields, including but not limited to: mitochondrial metabolism/disorders [17,19,20,21,22,23,24,25], cancer [26,27], bone metabolism [18], endocrinology [28,29], psychiatric disorders [30], inflammatory bowel disease [31], viral infections [32,33,34,35], allergies [36,37], circadian rhythms [38], and pain research [39].

## 4. Conclusions

We validated our high-throughput targeted and semiquantitative analytical method according to the EMA guidelines for bioanalytical methods. The key features of our method include,

(i) Optimization: well-characterized protocols for various biological matrices from different organisms enabled to study wide variety of research projects.
(ii) Accuracy/Precision: the targeted and semiquantitative analysis using 102 external 11-point calibration curves, including 12 labeled internal standards in every analysis, made it possible to compare the data within and between the studies.
(iii) Quality management: standard operating protocols, good laboratory practices, strict quality-management system, and proper documentation using electronic laboratory notebook enabled to check/retrieve very old data.
(iv) High-throughput: automated sample preparation and short analysis time (17.5 min), enabled high-throughput capabilities, which is the most desired feature for large-scale analyses.
(v) Stability: long-term stability studies in stock and intermediate solutions, wet extract, and freeze-thaw stability studies, critical for projects based on clinical/biobank samples.
(vi) Automation: downstream data processing steps in an automated manner reduces the interbatch variation and human errors, valuable for analyzing population cohorts and in epidemiological studies.
(vii) Reproducibility: low %CV of concentrations, retention times, correlation coefficient of calibration curves for an extended period of time, very important in metabolomics studies when comparing the data produced at different points of time.
(viii) Reliability: as shown by the excellent correlation between metabolite concentrations measured using our method and the NIST SRM plasma reference values, our method serves a standardised and reliable platform for metabolomics studies.
(ix) Robustness: Our results demonstrate an excellent cross-platform comparability with two completely different analytical platforms, a highly desirable criterion in multicenter studies when comparing the data across different laboratories using different instrumentation, protocols and analytical platforms.
(x) Data sharing: the huge QC sample database of healthy adults (N = 539) collected for six years and shared with the scientific community, provide normal reference values such as those provided in the HMDB database.

The above-mentioned aspects are critical in the metabolomics analysis, as the International Metabolomics Community has been putting a lot of efforts in data standardization, QC, reproducibility, robustness, and data sharing with the goal of moving towards applicability and integration of metabolomics data in precision/personalised medicine. In this context, our method provides a very timely and important contribution to the field. Furthermore, we have widely applied this method in many biomedical research projects, clinical trials, epidemiological studies, and in biomarker discovery.

## Figures and Tables

**Table 1:** Median concentration levels (μmol/L) measured in pooled healthy adult serum samples.

		Population Median (µmol/L)		
			95% Credibility Interval	
Class and Metabolite Name	HMDB Id	Estimate	Lower	Upper
**1. Alpha Amino Acids and Derivatives**				
2-Aminoisobutyrate	HMDB0001906	1.128	0.809	1.509
4-l-Hydroxyproline	HMDB0000725	15.895	11.300	21.184
5-Hydroxytryptophan	HMDB0000472	0.043	0.031	0.058
ADMA	HMDB0001539	0.963	0.248	2.230
Alanine	HMDB0000161	477.946	339.667	635.383
Aminoadipate	HMDB0000510	2.121	1.502	2.818
Arginine	HMDB0000517	84.902	60.795	113.172
Asparagine	HMDB0000168	47.204	33.635	62.727
Aspartate	HMDB0000191	26.932	18.786	35.424
Betaine	HMDB0000043	100.045	72.251	134.758
Citrulline	HMDB0000904	27.815	19.812	36.972
Creatine	HMDB0000064	61.180	44.481	82.161
Creatinine	HMDB0000562	66.085	47.095	88.066
Cystathionine	HMDB0000099	0.131	0.093	0.174
Dimethylglycine	HMDB0000092	3.541	2.521	4.733
GABA	HMDB0000112	0.195	0.138	0.259
G-Glutamylcysteine	HMDB0001049	2.966	2.116	3.946
Glutamate	HMDB0000148	53.446	37.691	70.613
Glutamine	HMDB0000641	791.142	560.999	1050.605
Glutathione	HMDB0000125	0.021	0.015	0.028
Glycine	HMDB0000123	243.717	172.929	325.417
Guanidoacetate	HMDB0000128	2.635	1.880	3.498
Histidine	HMDB0000177	88.616	62.718	117.848
Homocysteine	HMDB0000742	0.480	0.124	1.153
Homoserine	HMDB0000719	0.337	0.240	0.448
Hydroxykynurenine	HMDB0000732	0.096	0.068	0.128
Isoleucine	HMDB0000172	83.316	58.352	110.232
Kynurenine	HMDB0000684	1.146	0.813	1.520
Leucine	HMDB0000687	126.072	90.139	167.353
Lysine	HMDB0000182	176.519	127.474	236.393
Methionine	HMDB0000696	29.201	21.081	39.249
Ornithine	HMDB0000214	90.177	65.043	120.796
Phenylalanine	HMDB0000159	88.326	63.916	117.977
Proline	HMDB0000162	251.689	180.529	335.079
SDMA	HMDB0003334	2.862	2.060	3.834
Serine	HMDB0000187	148.482	107.012	198.507
Threonine	HMDB0000167	152.013	108.810	203.651
Tryptophan	HMDB0000929	33.961	24.104	45.261
Tyrosine	HMDB0000158	65.558	45.778	86.262
Valine	HMDB0000883	394.932	282.460	526.776
**2. Benzoic Acids and Derivatives**				
3-Hydroxanthranilate	HMDB0001476	0.188	0.134	0.251
Hippurate	HMDB0000714	6.101	4.362	8.124
**3. Beta Amino Acids and Derivatives**				
Carnosine	HMDB0000033	0.014	0.010	0.019
Pantothenate	HMDB0000210	0.310	0.220	0.411
**4. Bile Acids, Alcohols and Derivatives**				
Chenodeoxycholate	HMDB0000518	53.661	38.588	71.987
Cholate	HMDB0000619	0.676	0.483	0.900
Glycocholate	HMDB0000138	0.373	0.267	0.498
Taurochenodesoxycholate	HMDB0000951	0.507	0.359	0.673
**5. Carbohydrates and Carbohydrate Conjugates**				
d-Ribose-5-P	HMDB0001548	1.273	0.919	1.710
Glyceraldehyde	HMDB0001051	239.946	172.630	322.782
Sucrose	HMDB0000258	1.417	1.015	1.886
**6. Dialkylamines**				
Spermidine	HMDB0001257	33.601	23.958	44.759
**7. Dicarboxylic Acids and Derivatives**				
Succinate	HMDB0000254	7.912	5.597	10.491
**8. Fatty Acyls**				
Acetylcarnitine	HMDB0000201	9.709	2.323	22.264
Decanoylcarnitine	HMDB0000651	0.305	0.087	0.720
Hexanoylcarnitine	HMDB0000705	0.055	0.015	0.129
Isobutyrylcarnitine	HMDB0000736	0.248	0.061	0.578
Isovalerylcarnitine	HMDB0000688	0.102	0.027	0.240
Octanoylcarnitine	HMDB0000791	0.297	0.076	0.691
Propionylcarnitine	HMDB0000824	0.423	0.119	0.998
**9. Folates**				
Folate	HMDB0000121	0.011	0.003	0.027
**10. Glucuronic Acid and Derivatives**				
Glucuronate	HMDB0000127	1.960	1.404	2.627
**11. Imidazoles**				
1-Methylhistamine	HMDB0000898	0.006	0.004	0.008
Allantoin	HMDB0000462	2.447	1.741	3.254
**12. Indoles and Derivatives**				
5-Hydroxyindoleacetate	HMDB0000763	0.074	0.053	0.099
**13. Keto Acids and Derivatives**				
Acetoacetate	HMDB0000060	6.713	4.798	8.988
**14. Organic Phosphoric Acids and Derivatives**				
Phosphoethanolamine	HMDB0000224	3.316	2.356	4.420
**15. Organosulfonic Acids**				
Taurine	HMDB0000251	221.359	156.971	294.936
Taurocholate	HMDB0000036	0.083	0.060	0.112
**16. Oxides**				
Trimethylamine N-oxide	HMDB0000925	1.477	1.055	1.973
**17. Phenols**				
Homogentisate	HMDB0000130	0.115	0.082	0.153
Normetanephrine	HMDB0000819	0.0010	0.0007	0.0014
**18. Pteridines and Derivatives**				
Neopterin	HMDB0000845	0.005	0.004	0.007
**19. Purines and Derivatives**				
Adenine	HMDB0000034	0.007	0.005	0.009
Adenosine	HMDB0000050	0.008	0.006	0.011
AMP	HMDB0000045	0.106	0.075	0.140
cAMP	HMDB0000058	0.005	0.003	0.006
cGMP	HMDB0001314	0.009	0.002	0.026
Guanosine	HMDB0000133	0.445	0.315	0.593
Hypoxanthine	HMDB0000157	58.838	41.908	78.087
IMP	HMDB0000175	0.212	0.151	0.282
Inosine	HMDB0000195	36.061	25.798	48.008
Xanthine	HMDB0000292	3.926	2.790	5.211
Xanthosine	HMDB0000299	0.352	0.249	0.466
**20. Pyridines and Derivatives**				
4-Pyridoxate	HMDB0000017	0.057	0.042	0.077
Cotinine	HMDB0001046	0.531	0.382	0.710
NAD	HMDB0000902	0.015	0.011	0.020
Niacinamide	HMDB0001406	0.395	0.279	0.524
Nicotinate	HMDB0001488	0.012	0.009	0.017
Pyridoxine	HMDB0000239	0.0007	0.0005	0.0009
**21. Pyrimidines and Derivatives**				
Cytidine	HMDB0000089	0.003	0.002	0.004
Cytosine	HMDB0000630	0.080	0.056	0.106
Deoxycytidine	HMDB0000014	0.871	0.440	1.461
Deoxyuridine	HMDB0000012	0.543	0.391	0.726
Orotate	HMDB0000226	0.036	0.006	0.096
UDP Glucose	HMDB0000286	0.232	0.165	0.308
Uracil	HMDB0000300	0.058	0.042	0.078
**22. Quaternary Ammonium Salts**				
Carnitine	HMDB0000062	85.669	61.210	114.060
Choline	HMDB0000097	95.170	67.565	126.279
**23. Quinolines and Derivatives**				
Kynurenate	HMDB0000715	0.044	0.031	0.058
**24. Sugar Alcohols**				
Myo-inositol	HMDB0000211	16.989	12.075	22.606
Sorbitol	HMDB0000247	3.692	2.617	4.904

## Supplementary Materials

The following are available online at http://www.mdpi.com/2218-1989/8/3/44/s1, Table S1: List of metabolites showing molecular weight, retention time, linearity of calibration, and compound dependent MS parameters and catalogue numbers. Supplementary text: A detailed method description. Figure S1: Chromatograms for all the 102 metabolites of QC sample.
