# Supplementary material for: Validation and Automation of a High-Throughput Multitargeted Method for Semiquantification of Endogenous Metabolites from Different Biological Matrices Using Tandem Mass Spectrometry
Source: Metabolites. 2018 Aug 5;8(3):44. doi: 10.3390/metabo8030044 (PMC6161248; doi:10.3390/metabo8030044)
Supplement: Supplementary file 1 [file metabolites-08-00044-s001.zip › Supp Fig 1_19072018.pdf]

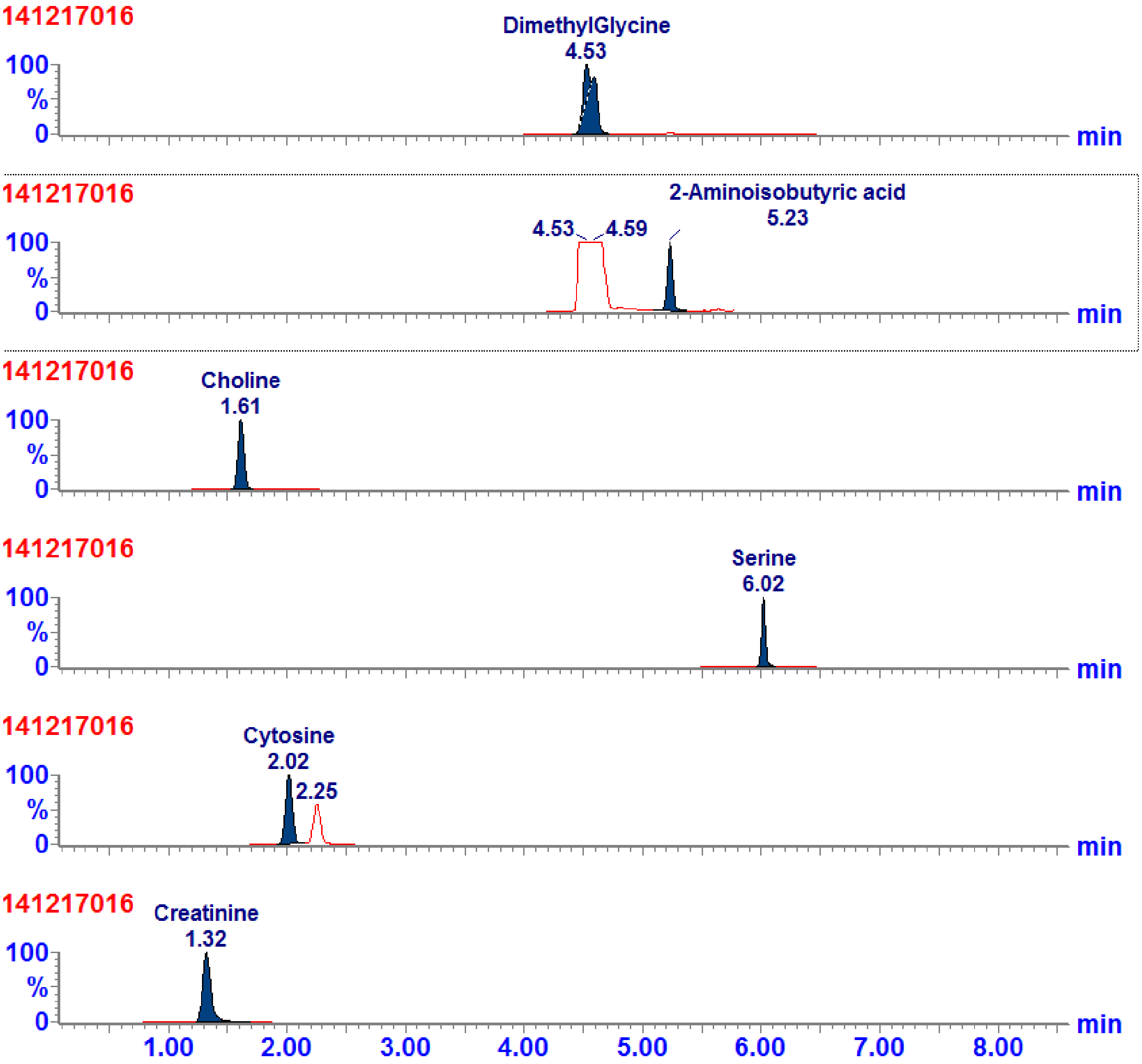
141217016
DimethylGlycine
4.53
100
%
0
min
141217016
2-Aminoisobutyric acid
5.23
4.53
4.59
100
%
0
min
141217016
Choline
1.61
100
%
0
min
141217016
Serine
6.02
100
%
0
min
141217016
Cytosine
2.02
2.25
100
%
0
min
141217016
Creatinine
1.32
100
%
0
min
1.00
2.00
3.00
4.00
5.00
6.00
7.00
8.00

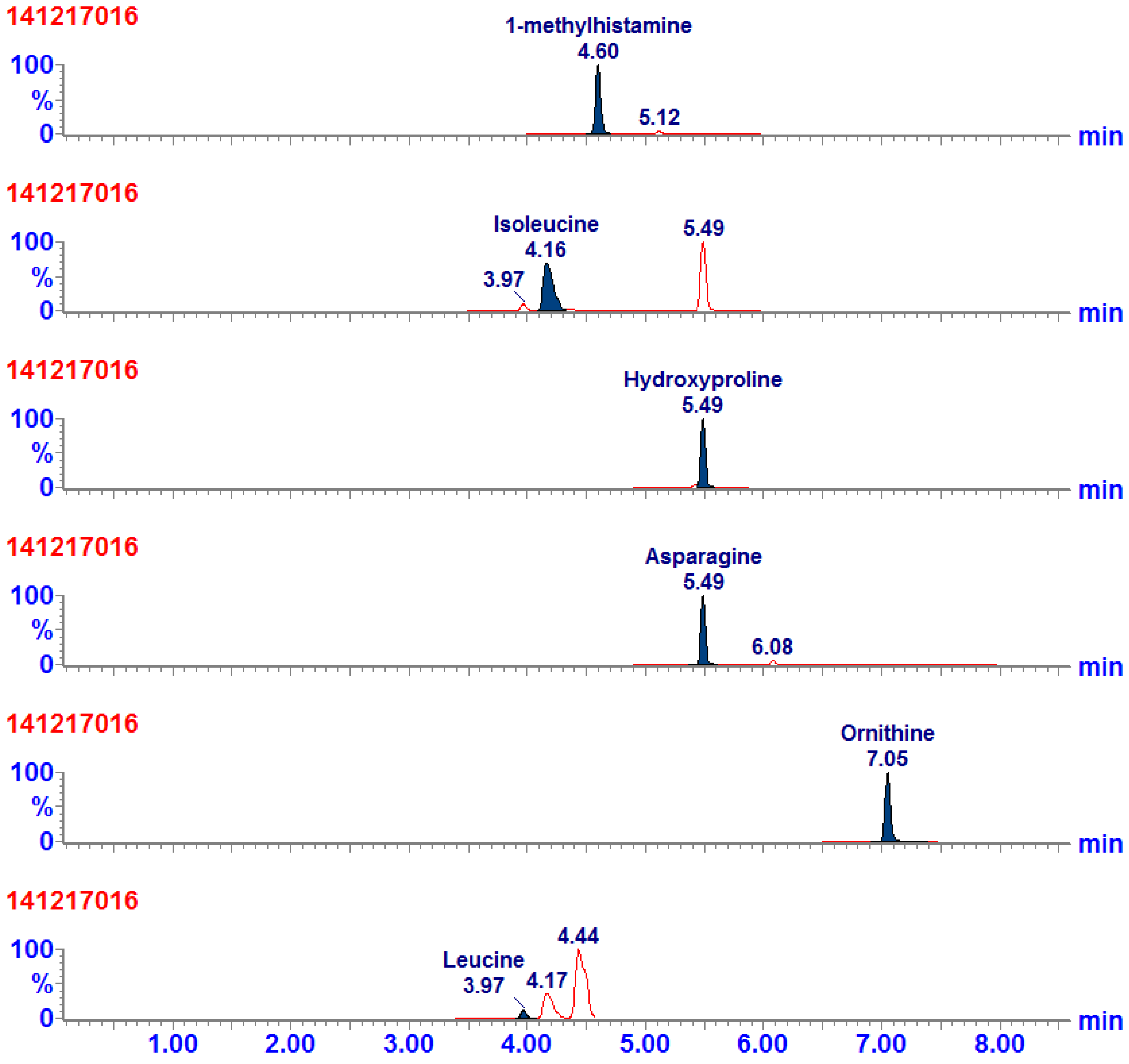

141217016
1-methylhistamine
4.60
5.12
141217016
Isoleucine
4.16
3.97
5.49
141217016
Hydroxyproline
5.49
141217016
Asparagine
5.49
6.08
141217016
Ornithine
7.05
141217016
Leucine
3.97
4.17
4.44
100
%
0
min
1.00
2.00
3.00
4.00
5.00
6.00
7.00
8.00

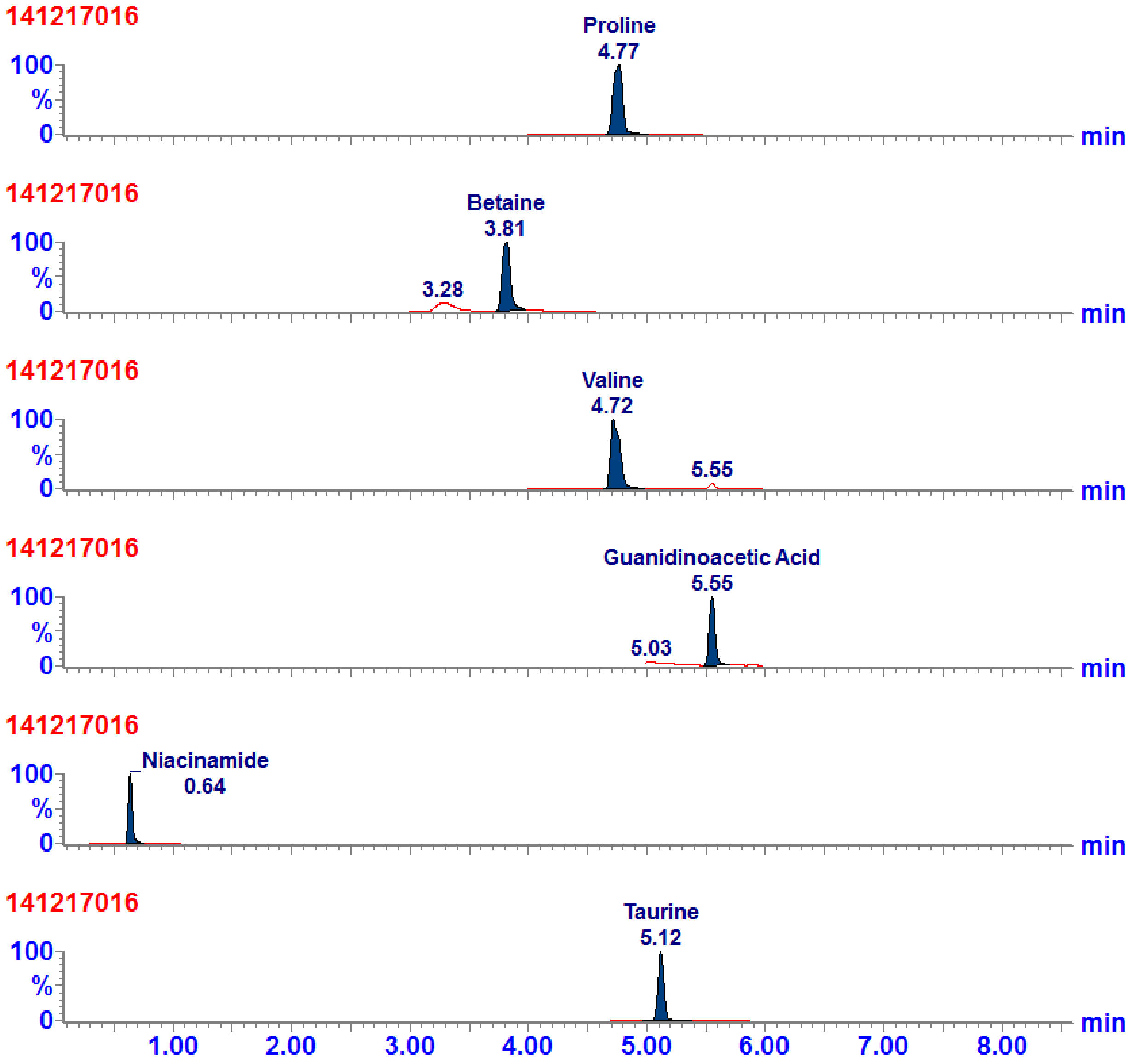
141217016
Proline
4.77
100
%
0
min
141217016
Betaine
3.81
3.28
100
%
0
min
141217016
Valine
4.72
5.55
100
%
0
min
141217016
Guanidinoacetic Acid
5.55
5.03
100
%
0
min
141217016
Niacinamide
0.64
100
%
0
min
141217016
Taurine
5.12
100
%
0
min
1.00
2.00
3.00
4.00
5.00
6.00
7.00
8.00

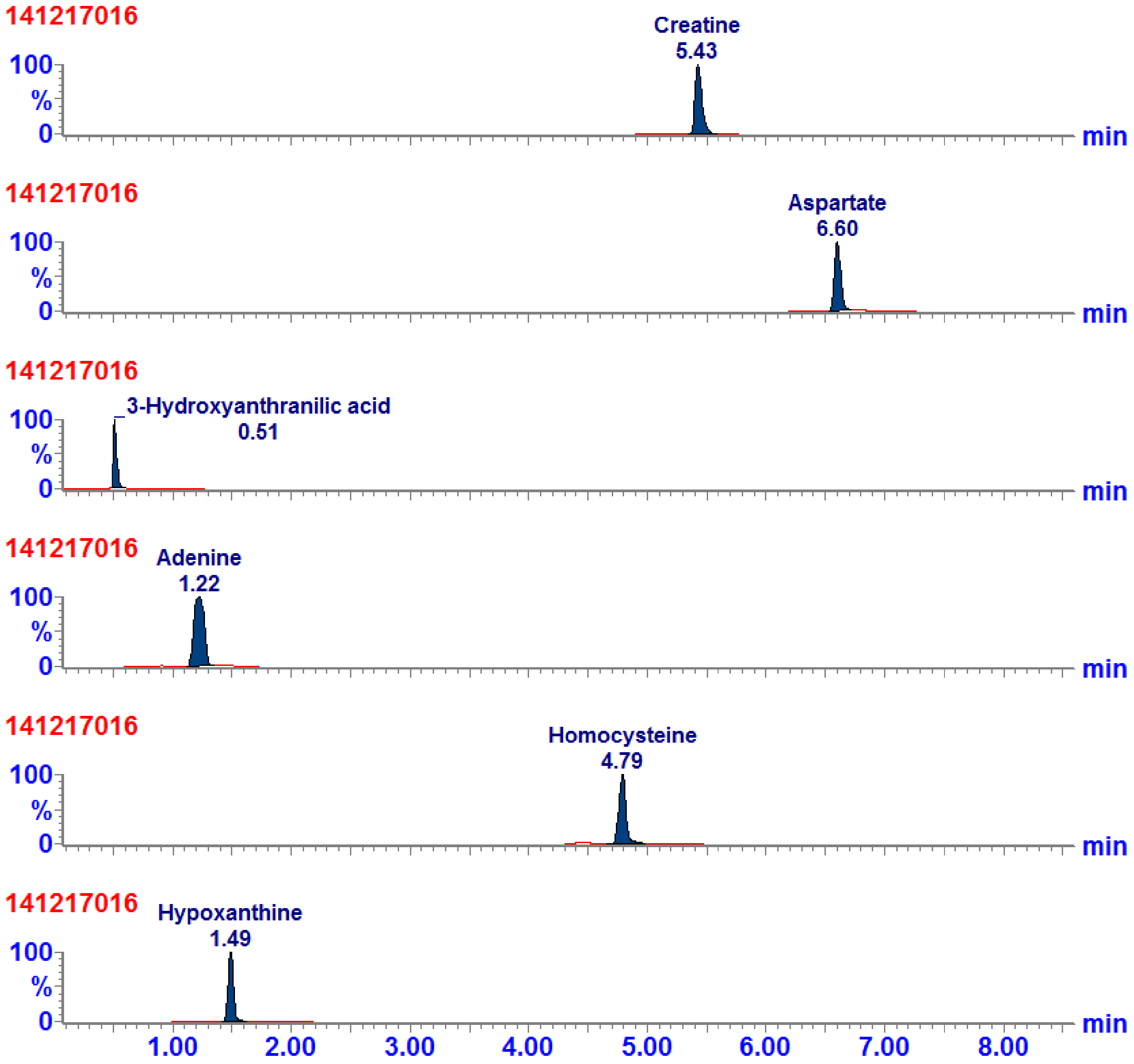
141217016
Creatine
5.43
100
%
0
min
141217016
Aspartate
6.60
100
%
0
min
141217016
3-Hydroxyanthranilic acid
0.51
100
%
0
min
141217016
Adenine
1.22
100
%
0
min
141217016
Homocysteine
4.79
100
%
0
min
141217016
Hypoxanthine
1.49
100
%
0
min
1.00
2.00
3.00
4.00
5.00
6.00
7.00
8.00

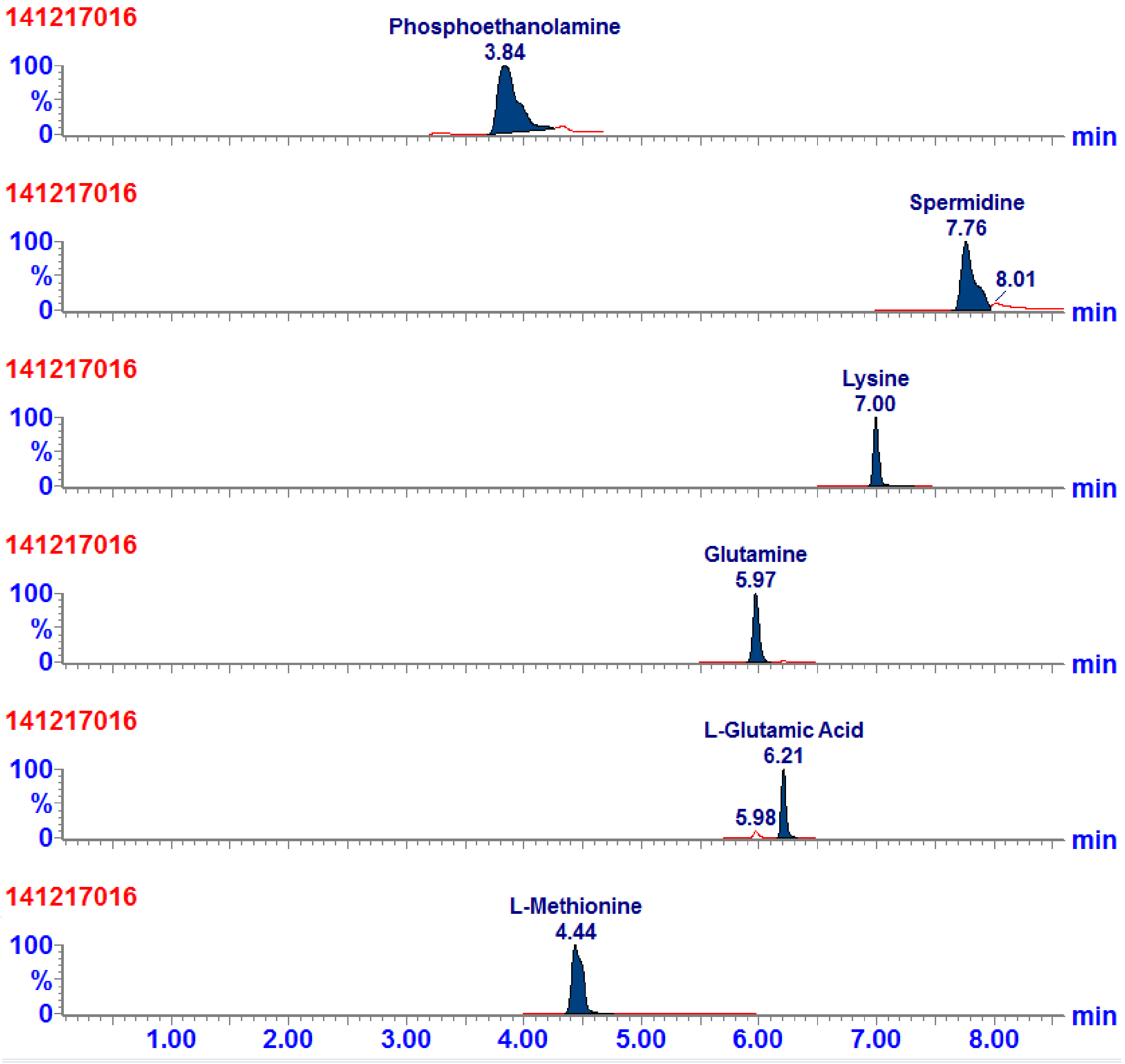
141217016
Phosphoethanolamine
3.84
100
%
0
min
141217016
Spermidine
7.76
8.01
100
%
0
min
141217016
Lysine
7.00
100
%
0
min
141217016
Glutamine
5.97
100
%
0
min
141217016
L-Glutamic Acid
6.21
5.98
100
%
0
min
141217016
L-Methionine
4.44
100
%
0
1.00
2.00
3.00
4.00
5.00
6.00
7.00
8.00
min

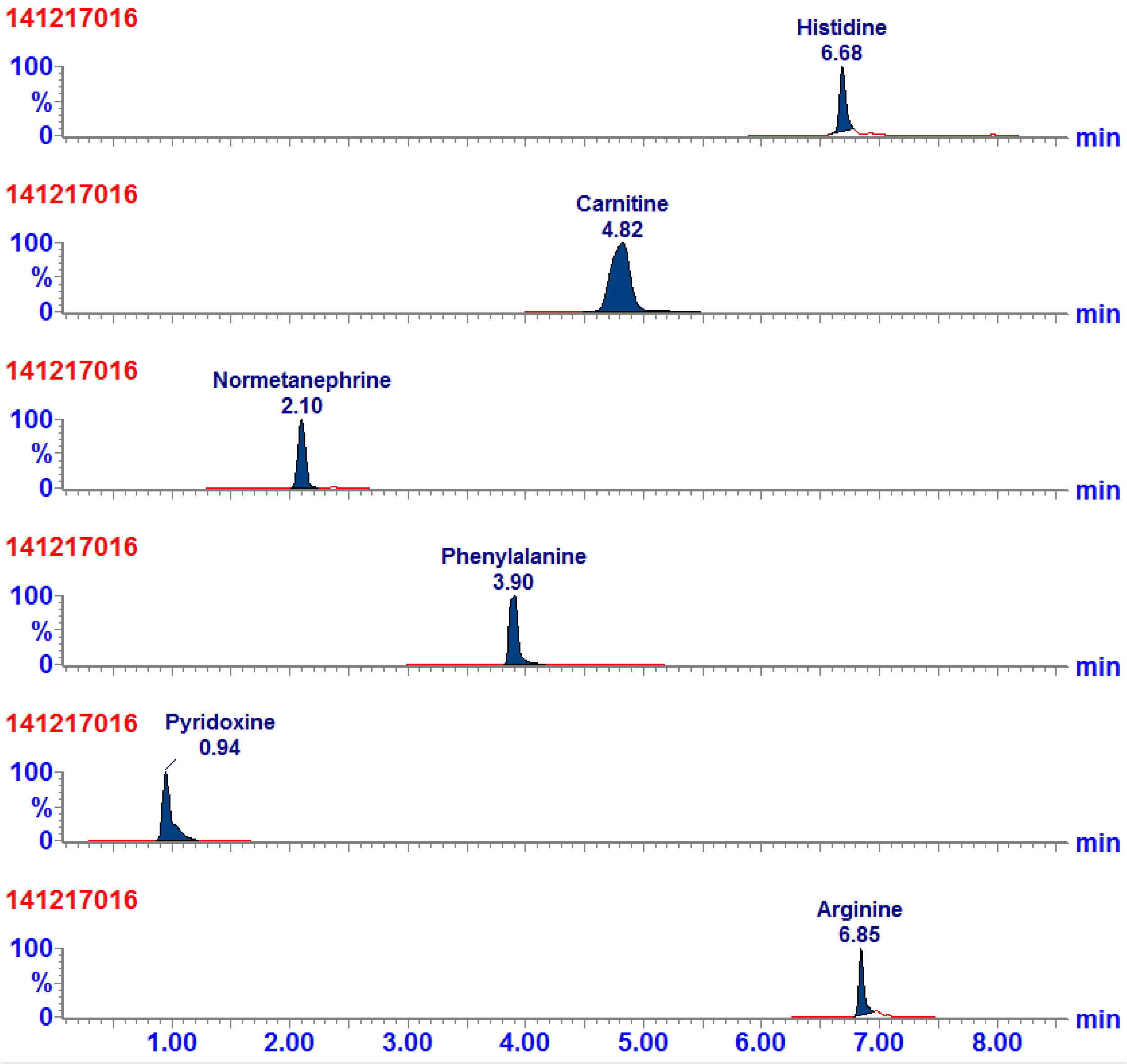
141217016
Histidine
6.68
141217016
Carnitine
4.82
141217016
Normetanephrine
2.10
141217016
Phenylalanine
3.90
141217016
Pyridoxine
0.94
141217016
Arginine
6.85
100
%
0
min
1.00
2.00
3.00
4.00
5.00
6.00
7.00
8.00

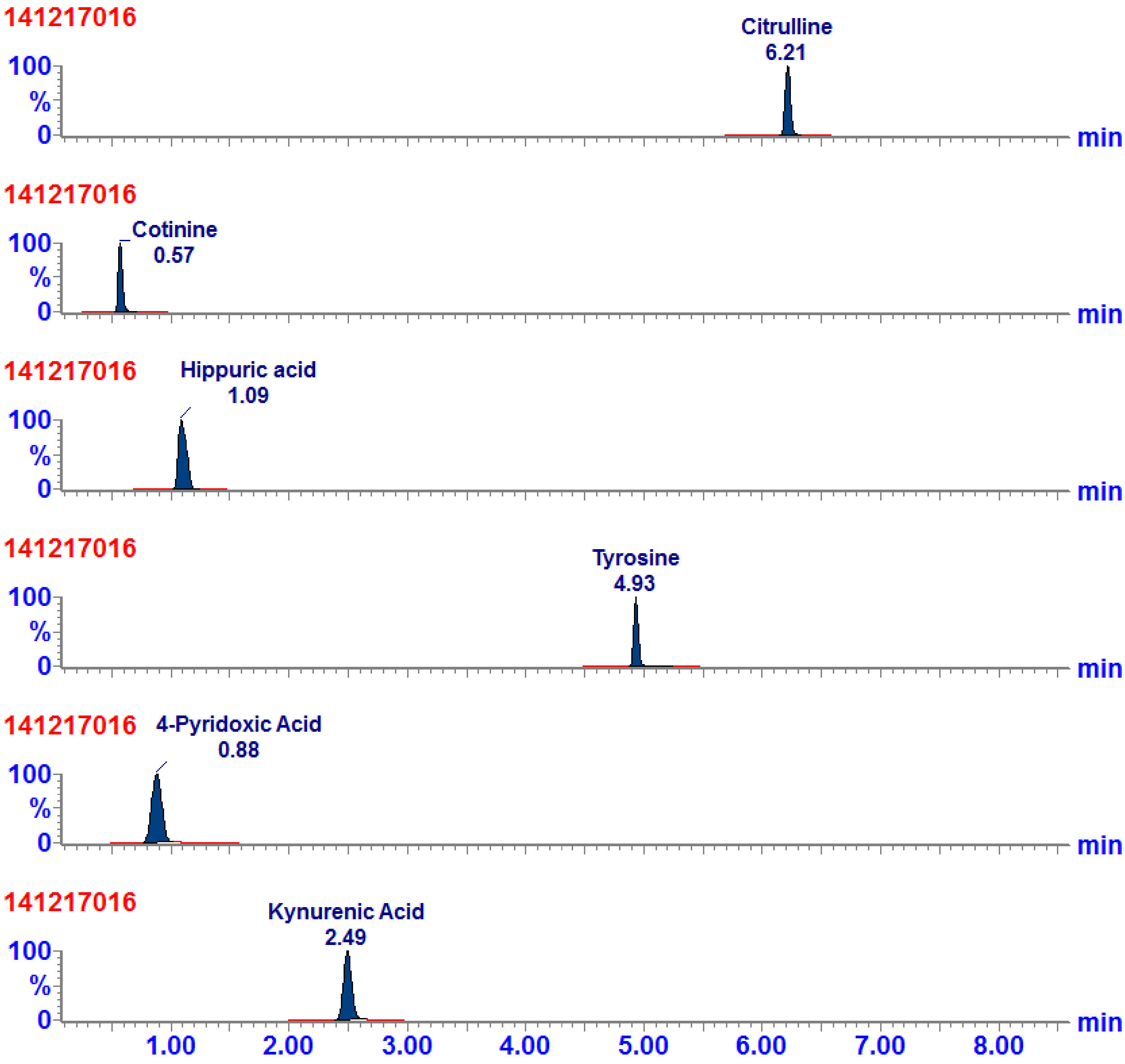
141217016
Citrulline
6.21
100
%
0
min
141217016
Cotinine
0.57
100
%
0
min
141217016
Hippuric acid
1.09
100
%
0
min
141217016
Tyrosine
4.93
100
%
0
min
141217016
4-Pyridoxic Acid
0.88
100
%
0
min
141217016
Kynurenic Acid
2.49
100
%
0
min
1.00
2.00
3.00
4.00
5.00
6.00
7.00
8.00

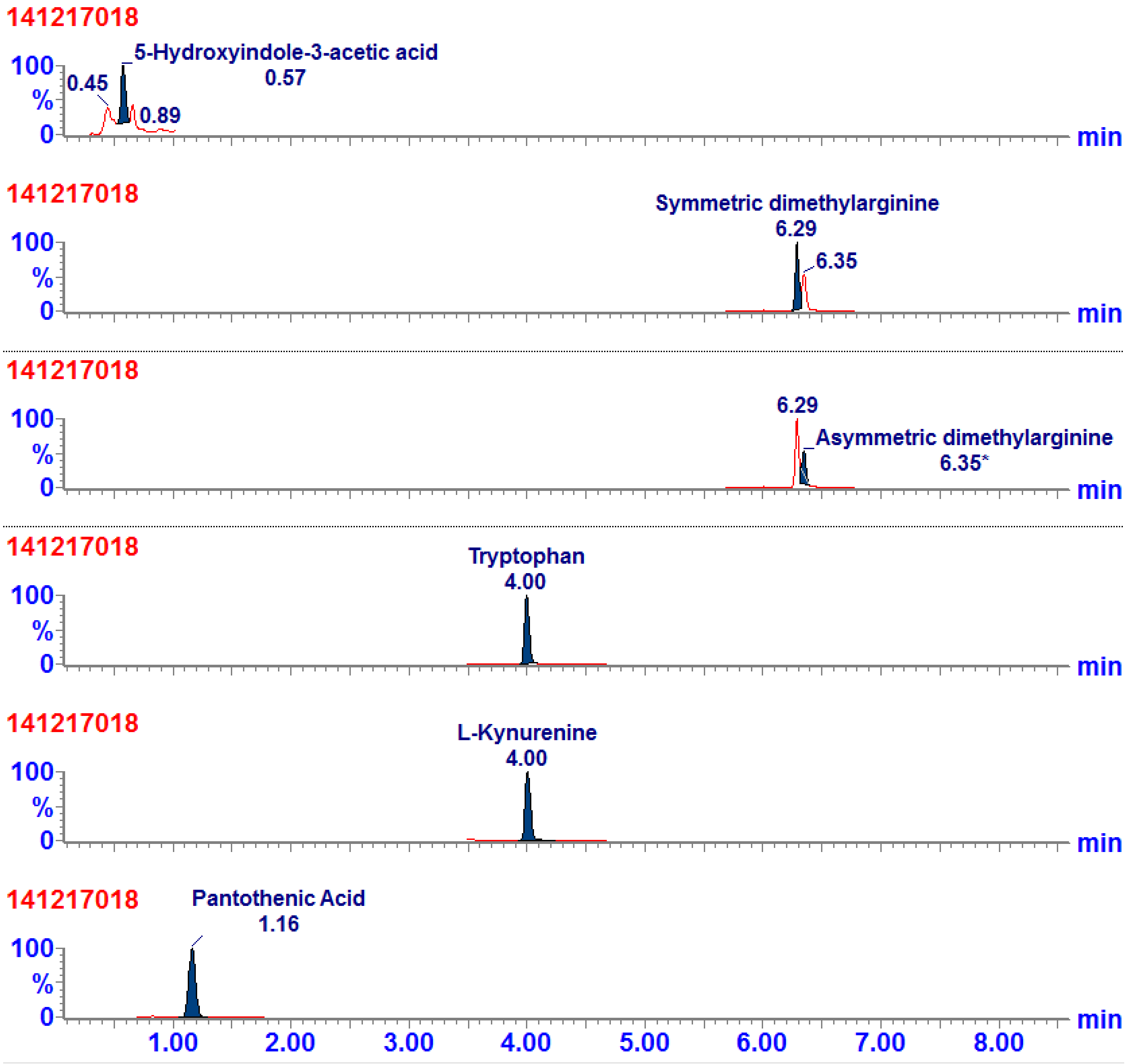
141217018
5-Hydroxyindole-3-acetic acid
0.57
0.45
0.89
141217018
Symmetric dimethylarginine
6.29
6.35
141217018
6.29
Asymmetric dimethylarginine
6.35*
141217018
Tryptophan
4.00
141217018
L-Kynurenine
4.00
141217018
Pantothenic Acid
1.16
100
%
0
min
1.00
2.00
3.00
4.00
5.00
6.00
7.00
8.00

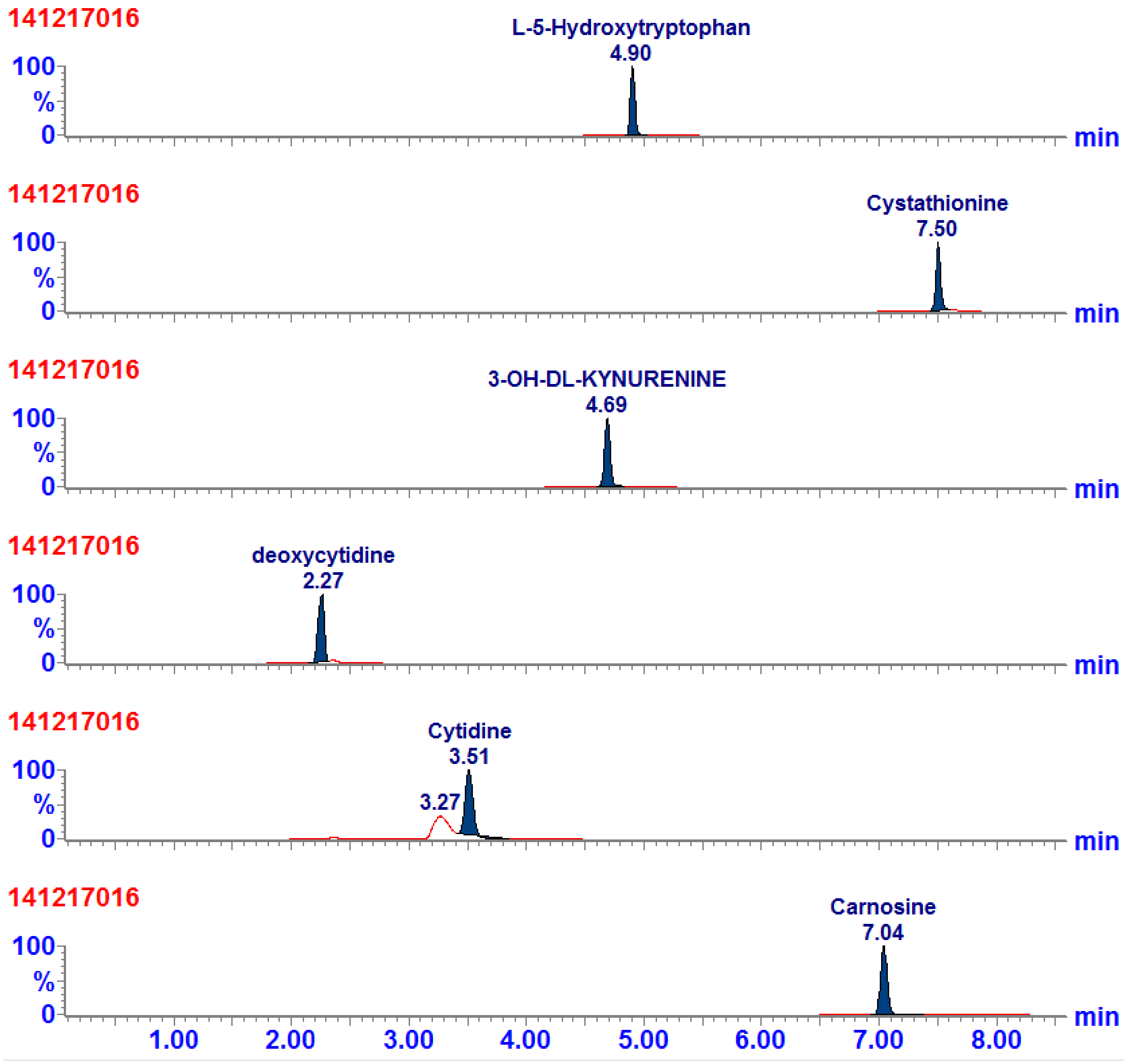
141217016
L-5-Hydroxytryptophan
4.90
141217016
Cystathionine
7.50
141217016
3-OH-DL-KYNURENINE
4.69
141217016
deoxycytidine
2.27
141217016
Cytidine
3.51
3.27
141217016
Carnosine
7.04
100
%
0
min
1.00
2.00
3.00
4.00
5.00
6.00
7.00
8.00

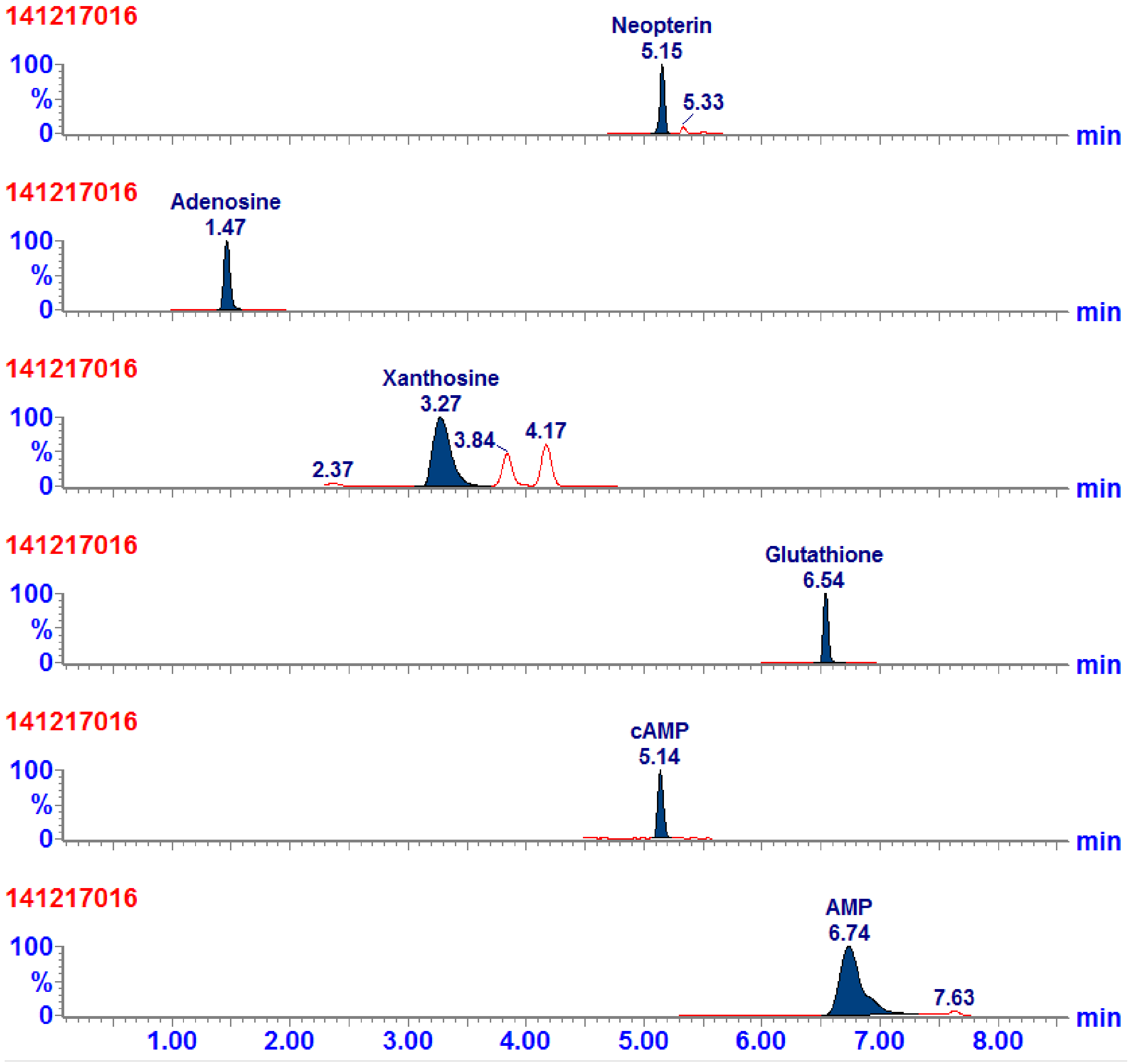
141217016
Neopterin
5.15
5.33
100
%
0
min
141217016
Adenosine
1.47
100
%
0
min
141217016
Xanthosine
3.27
3.84
4.17
2.37
100
%
0
min
141217016
Glutathione
6.54
100
%
0
min
141217016
cAMP
5.14
100
%
0
min
141217016
AMP
6.74
7.63
100
%
0
min
1.00
2.00
3.00
4.00
5.00
6.00
7.00
8.00

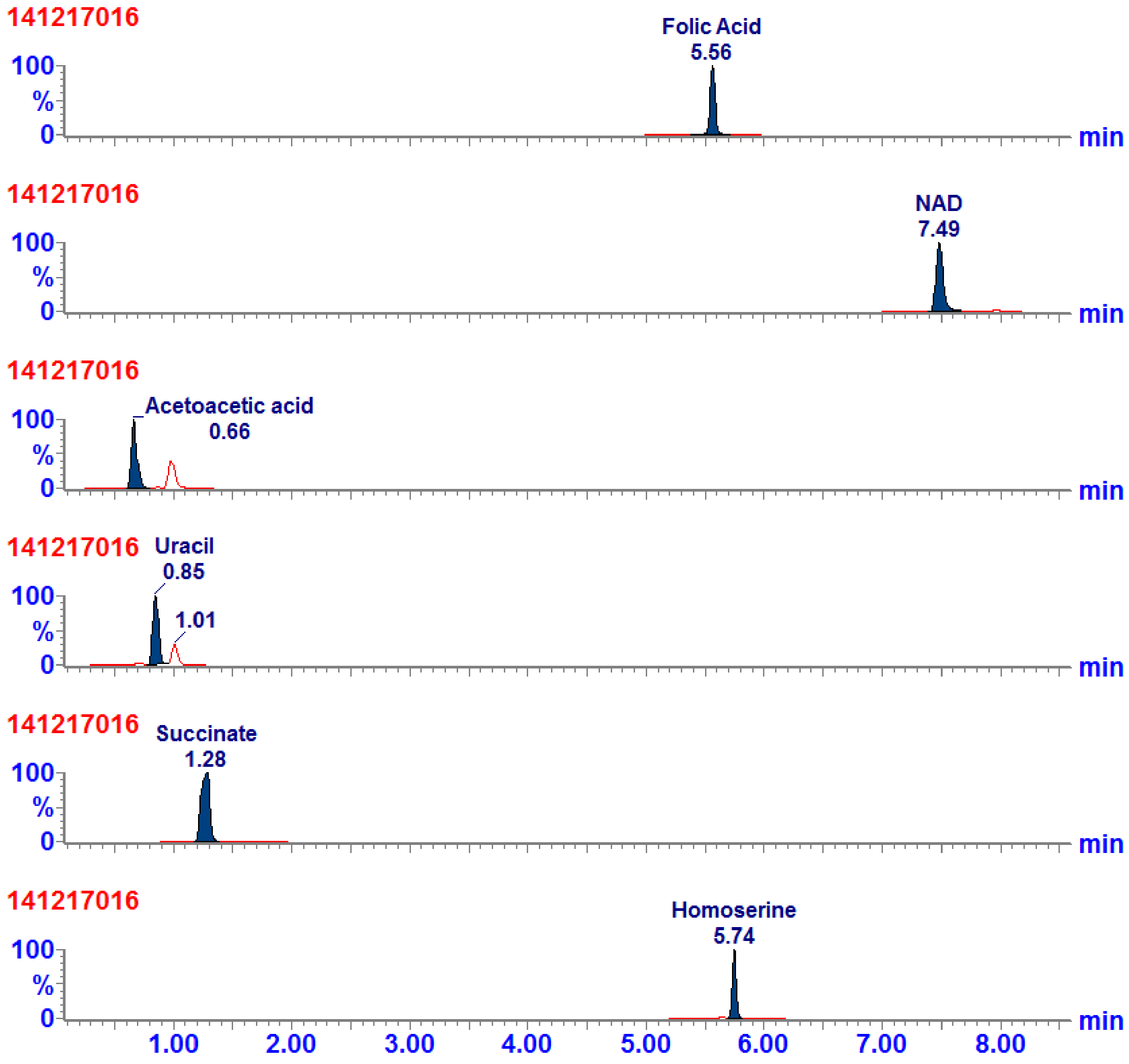
141217016
Folic Acid
5.56
100
%
0
min
141217016
NAD
7.49
100
%
0
min
141217016
Acetoacetic acid
0.66
100
%
0
min
141217016
Uracil
0.85
1.01
100
%
0
min
141217016
Succinate
1.28
100
%
0
min
141217016
Homoserine
5.74
100
%
0
min
1.00
2.00
3.00
4.00
5.00
6.00
7.00
8.00

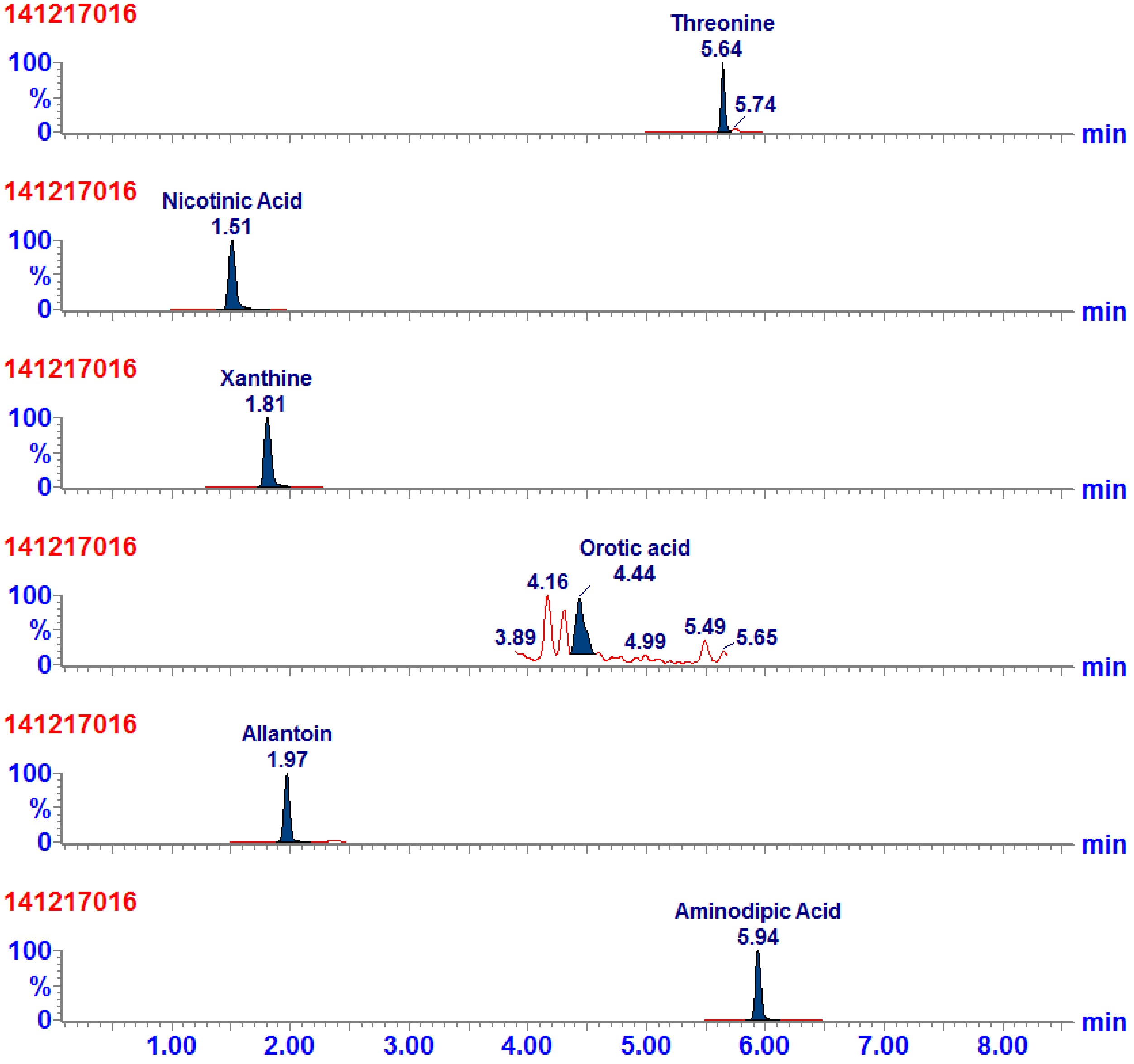
141217016
Threonine
5.64
5.74
100
%
0
min
141217016
Nicotinic Acid
1.51
100
%
0
min
141217016
Xanthine
1.81
100
%
0
min
141217016
Orotic acid
4.16
4.44
3.89
4.99
5.49
5.65
100
%
0
min
141217016
Allantoin
1.97
100
%
0
min
141217016
Aminodipic Acid
5.94
100
%
0
min
1.00
2.00
3.00
4.00
5.00
6.00
7.00
8.00

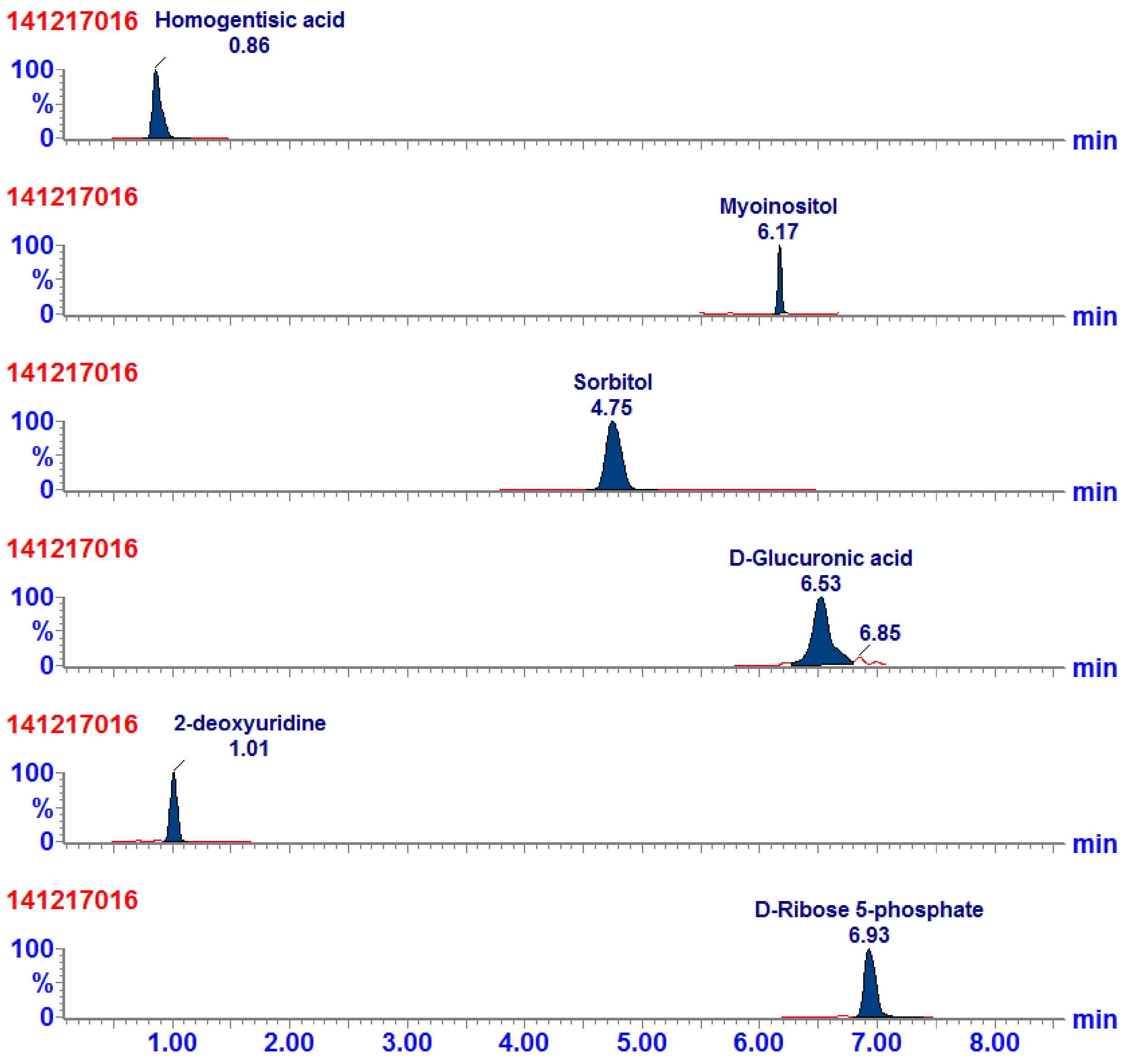

141217016
Homogentisic acid
0.86
141217016
Myoinositol
6.17
141217016
Sorbitol
4.75
141217016
D-Glucuronic acid
6.53
6.85
141217016
2-deoxyuridine
1.01
141217016
D-Ribose 5-phosphate
6.93
100
%
0
min
1.00
2.00
3.00
4.00
5.00
6.00
7.00
8.00

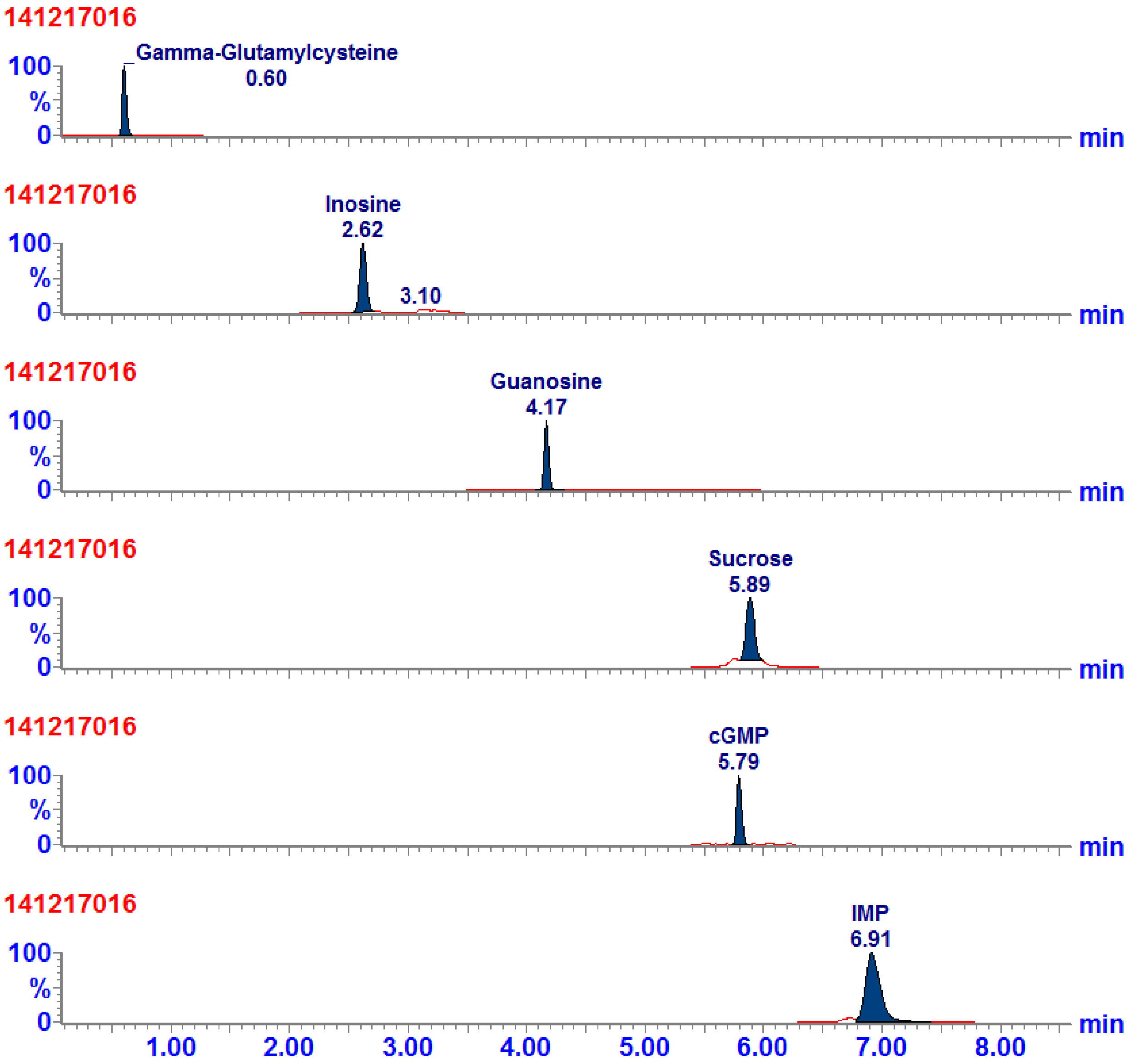
141217016
Gamma-Glutamylcysteine
0.60
100
%
0
min
141217016
Inosine
2.62
3.10
100
%
0
min
141217016
Guanosine
4.17
100
%
0
min
141217016
Sucrose
5.89
100
%
0
min
141217016
cGMP
5.79
100
%
0
min
141217016
IMP
6.91
100
%
0
min
1.00
2.00
3.00
4.00
5.00
6.00
7.00
8.00

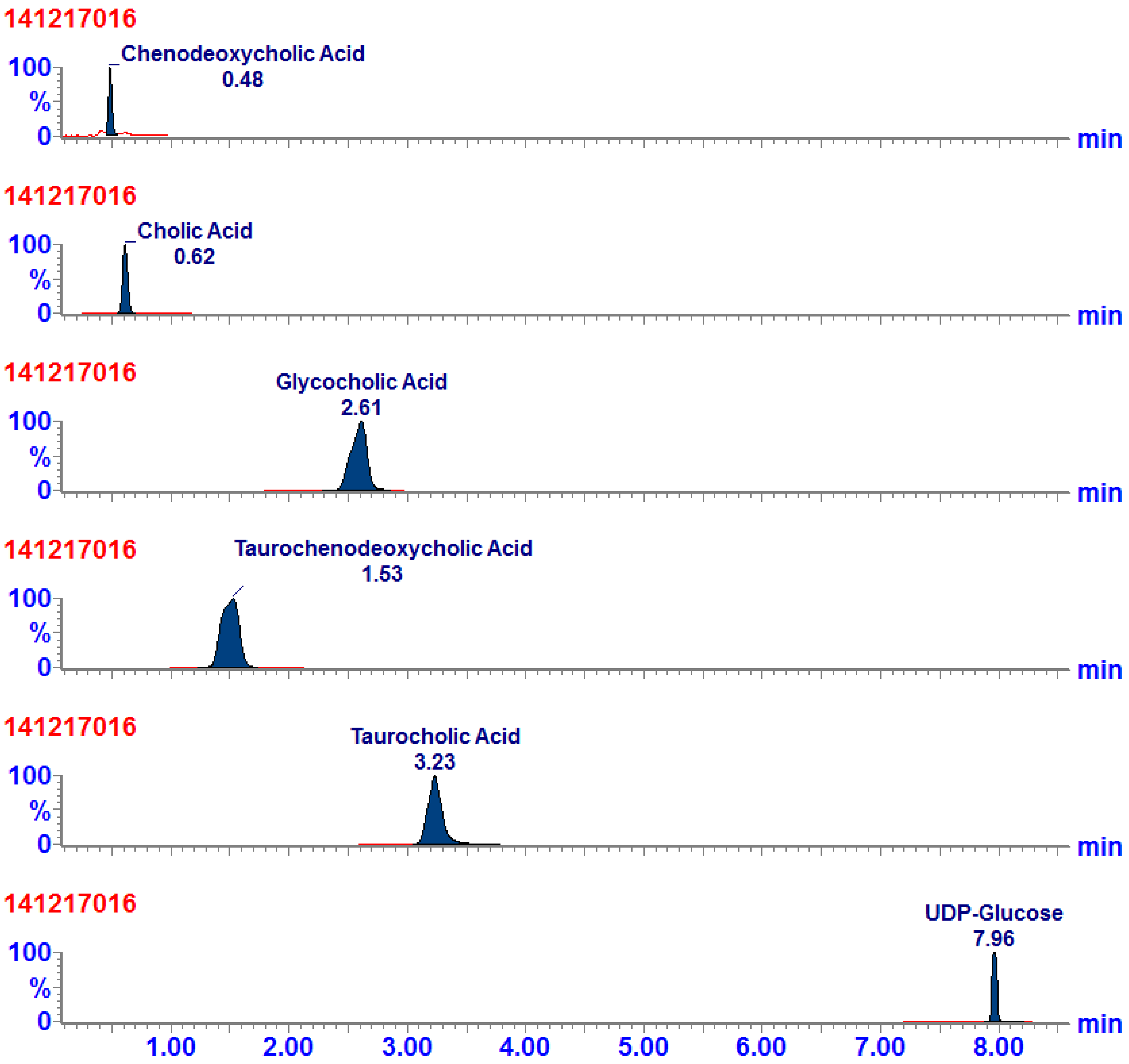
141217016
Chenodeoxycholic Acid
0.48
141217016
Cholic Acid
0.62
141217016
Glycocholic Acid
2.61
141217016
Taurochenodeoxycholic Acid
1.53
141217016
Taurocholic Acid
3.23
141217016
UDP-Glucose
7.96
100
%
0
min
1.00
2.00
3.00
4.00
5.00
6.00
7.00
8.00

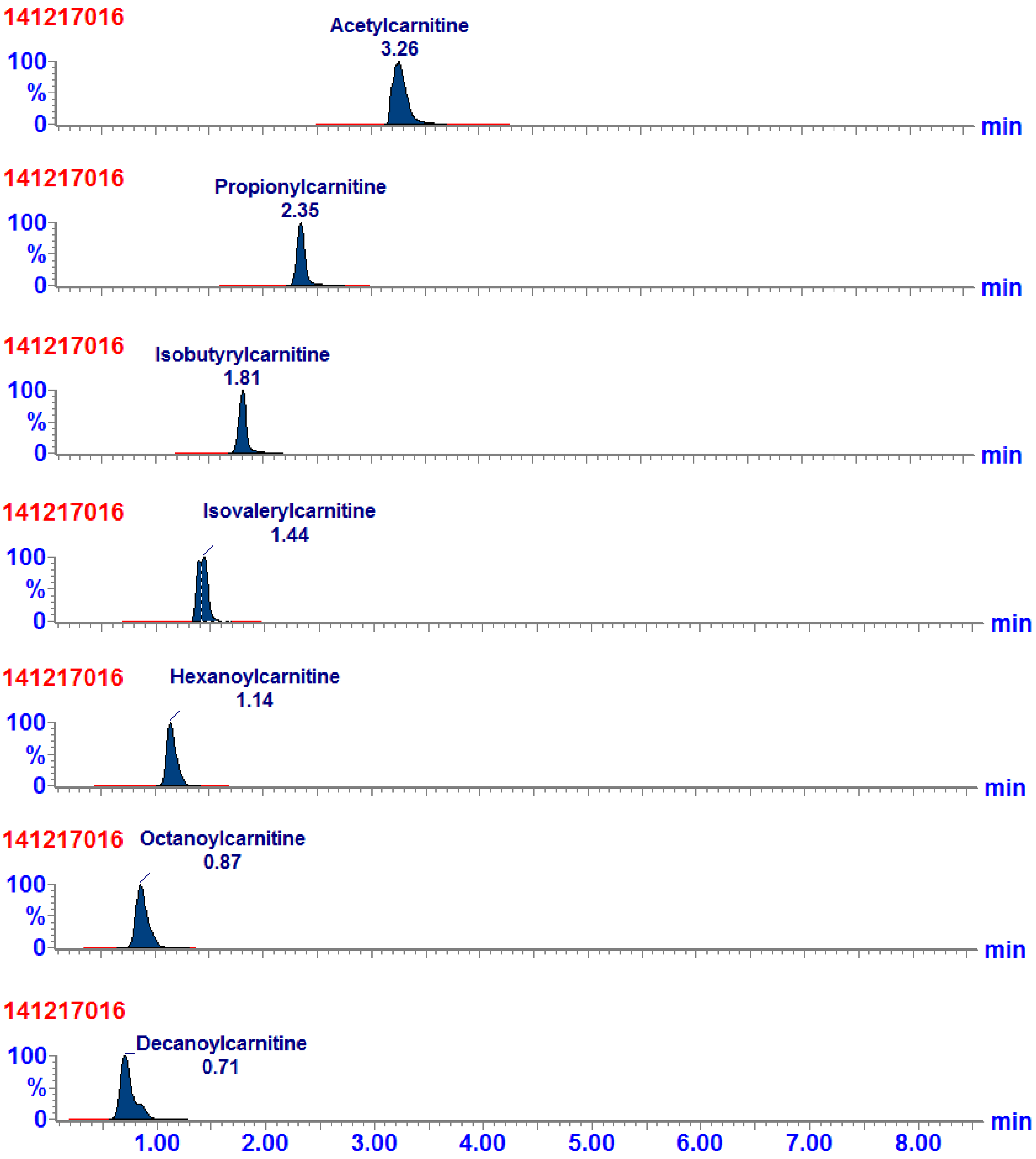
141217016
Acetylcarnitine
3.26
141217016
Propionylcarnitine
2.35
141217016
Isobutyrylcarnitine
1.81
141217016
Isovalerylcarnitine
1.44
141217016
Hexanoylcarnitine
1.14
141217016
Octanoylcarnitine
0.87
141217016
Decanoylcarnitine
0.71
100
%
0
min
1.00
2.00
3.00
4.00
5.00
6.00
7.00
8.00
