# Supplementary material for: Validation and Automation of a High-Throughput Multitargeted Method for Semiquantification of Endogenous Metabolites from Different Biological Matrices Using Tandem Mass Spectrometry
Source: Metabolites. 2018 Aug 5;8(3):44. doi: 10.3390/metabo8030044 (PMC6161248; doi:10.3390/metabo8030044)
Supplement: Supplementary file 1 [file metabolites-08-00044-s001.zip › Supp method description_19072018.docx]

**Tissue samples extraction:**

Frozen tissue samples were weighed (20±5 mg) and transferred to Precellys homogenization tubes (Precellys 24 lysing kit, Precellys) containing 1.4 mm ceramic (Zirconium oxide) beads followed by addition of 20 uL of labeled internal standard mix and incubated on ice for 10 min. After incubation, homogenizations step was performed with a Precellys 24 homogenizer in a two-step extraction process. In the first step, 500 µL of precooled 99% ACN + 1% FA was added to the sample and homogenized for 3 cycles of 20 sec each at 5,500 rpm with 10 sec pause between each homogenization interval. After homogenization, the sample tubes were centrifuged for 10 min at 14,000 rpm at 4^o^C and the supernatant was collected in a 1.5 ml eppendorf tube. In the second step, 500 µL of 90/10% ACN/H_2_O + 1% FA was added to the remaining pellet and repeated the steps as above and finally pooled to the previous extract. The collected supernatant was dispensed into an OstroTM 96-well plate (Waters Corporation, Milford, USA) and then filtered by applying a vacuum at a delta pressure of 300–400 mbar for 2.5 min on a Hamilton robot's vacuum station. After this, 5 μL of filtered sample extract was injected into an Acquity UPLC system coupled to a Xevo® TQ-S triple quadrupole mass spectrometer (Waters Corporation, Milford, MA, USA).

**Cells pellet samples extraction:**

Around one million frozen cell samples were thawed step wise at -20^o^C and 4^o^C and then metabolites were extracted by adding 20 µL of labeled internal standard mix and 1 ml of cold extraction solvent (90/10 ACN/H_2_O + 1% FA). Cells were then sonicated for 30 sec, vortexed for 30 sec, and incubated on ice for 10 min. These steps of sonicating and vortexing were repeated for three times. After that sample tubes were centrifuged for 10 min at 14,000 rpm at 4^o^C and the supernatant was collected in a 1.5 ml eppendorf tube. The collected supernatant was dispensed into an OstroTM 96-well plate (Waters Corporation, Milford, USA) and then filtered by applying a vacuum at a delta pressure of 300–400 mbar for 2.5 min on a Hamilton robot's vacuum station. After this, 5 μL of filtered sample extract was injected into an Acquity UPLC system coupled to a Xevo® TQ-S triple quadrupole mass spectrometer (Waters Corporation, Milford, MA, USA).

**Biofluid samples extraction:**

Ten microliters of labeled internal standard mixture was added to 100 µL of biofluid sample and the samples were allowed to equilibrate with the internal standards. A total of 400 µL of extraction solvent (1% formic acid in acetonitrile) was added for protein precipitation. After that samples were centrifuged at 14,000 rpm at 4^o^C for 15 min and supernatants were collected and dispensed into an OstroTM 96-well plate (Waters Corporation, Milford, USA), and then filtered by applying a vacuum at a delta pressure of 300–400 mbar for 2.5 min on a Hamilton robot's vacuum station. After this, 5 μL of filtered sample extract was injected into an Acquity UPLC system coupled to a Xevo® TQ-S triple quadrupole mass spectrometer (Waters Corporation, Milford, MA, USA).

**Instrumentation and analytical conditions:**

The final analysis for all metabolites were performed on an ACQUITY UPLC-MS/MS system (Waters Corporation, Milford, MA, USA). Chromatographic separation was done using 2.1 × 100 mm Acquity 1.7um BEH amide HILIC column (Waters Corporation, Milford, MA, USA), and temperature was maintained at 45°C. The total run time is 14.5 min including 2.5 min of equilibration step at a flow rate of 600 µL/min. Initially the gradient started with a 2.5 min isocratic step at 100% mobile phase B (ACN/ H_2_O, 90/10 (v/v), 20 mM ammonium formate, pH at 3), and then rising to 100% mobile phase A (ACN/H_2_O, 50/50 (v/v), ammonium formate, pH at 3) over the next 10 min and maintained for 2min at 100% A and finally equilibrated to the initial conditions for 2.5 min. An injection volume of 5 µL of sample extract was used and two cycles of 300 µL of strong wash (methanol/isopropanol/ACN/H_2_O, 25/25/25/25, 0.5% FA) and 900 µL of weak wash (methanol/isopropanol/ACN/H_2_O, 25/25/25/25, 0.5% ammonium hydroxide) and in addition 2 min of seal wash (90/10, methanol/H_2_O) were carried out. The auto-sampler was used to perform partial loop with needle overfill injections for the samples and standards and kept at 5 ^o^C. The detection system, a Xevo® TQ-S tandem triple quadrupole mass spectrometer (Waters, Milford, MA, USA), was operated in both positive and negative polarities with a polarity switching time of 20 msec. Electro spray ionization (ESI) was chosen as the ionization mode with a capillary voltage at 0.6 KV in both polarities. The source temperature and desolvation temperature of 120°C and 650°C, respectively, were maintained constantly throughout the experiment. Cone voltage and collision energy (CE) were optimized for each compound. High pure nitrogen and argon gas were used as desolvation gas (600 L/hr) and collision gas (0.15 ml/min), respectively. Multiple Reaction Monitoring (MRM) acquisition mode was selected for quantification of metabolites with individual span time of 0.1 sec given in their individual MRM channels. The dwell time was calculated automatically by the software based on the region of the retention time window, number of MRM functions and also depending on the number of data points required to form the peak. MassLynx 4.1 software was used for data acquisition, data handling and instrument control. Data processing was done using TargetLynx software and metabolites were quantified by using labeled internal standards and external calibration curves.
